# Efficient extraction of atrazine in real water samples using a biopolymer ZnO@chitosan nanocomposite-based solid-phase extraction cartridge and its quantification by liquid chromatography-tandem mass spectrometry

**DOI:** 10.1039/d6ra06356j

**Published:** 2026-09-16

**Authors:** Safina Kamboh, Syeda Sara Hassan, Saira Sidhu, Muhammad Raheel Bawani, Akbar Ali, Khalid Hussain Thebo, Sabry M. Attia, Ahmed Nadeem

**Affiliations:** a U.S-Pakistan Centre for Advanced Studies in Water (USPCAS-W), Mehran University of Engineering & Technology Jamshoro 76062 Pakistan sshassan.uspcasw@faculty.muet.edu.pk; b Department of Mining Engineering, Mehran University of Engineering & Technology Jamshoro 76062 Pakistan; c MIIT Key Laboratory of Critical Materials Technology for New Energy Conversion and Storage, State Key Laboratory of Urban Water Resource and Environment, School of Chemistry and Chemical Engineering, Harbin Institute of Technology Harbin 150001 PR China; d Department of Chemistry, Mirpur University of Science and Technology Mirpur Azad Jammu & Kashmir Pakistan khalidthebo@yahoo.com; e Department of Pharmacology and Toxicology, College of Pharmacy, King Saud University Riyadh 11451 Saudi Arabia

## Abstract

Atrazine, a persistent herbicide frequently detected in agricultural runoff and groundwater, requires low-cost methods for environmental monitoring and remediation. Herein, a biopolymer zinc oxide-decorated chitosan (ZnO@CS) nanocomposite was synthesized *via* a sol–gel method and employed as a solid-phase extraction (SPE) sorbent for atrazine removal. The as-prepared ZnO@CS nanocomposite was characterized by UV-visible spectroscopy, scanning electron microscopy (particle sizes approximately 30 to 61 nm), energy-dispersive spectroscopy, Fourier-transform infrared spectroscopy, X-ray diffraction, and zeta-potential analysis. The as-prepared ZnO@CS-based SPE cartridge was used for the extraction recovery of the atrazine pesticide from synthetic and real water samples, and it achieved 98% atrazine recovery from synthetic water under optimized conditions of 100 µg L^−1^, pH 9, 25 °C, 100 min, and 100 mL. The adsorption followed pseudo-first-order kinetics (*R*^2^ = 0.998) and fitted the Freundlich isotherm (*R*^2^ = 0.969), consistent with adsorption on a heterogeneous surface. LC-MS/MS quantification analysis showed a linearity of *R*^2^ = 0.992. Moreover, the as-prepared ZnO@CS-based SPE cartridge demonstrated high atrazine recovery values of up to 70% and 68% from real field water and groundwater, respectively, which are higher than that achieved with the commercial C18 cartridge (60%) for real field water. We believe that the as-prepared ZnO@CS-based nanocomposite can be a promising sorbent for atrazine extraction recovery in the future due to its cost-effectiveness, environmental friendliness, and superior extraction efficiency.

## Abstract 2

In this study, a zinc oxide-decorated chitosan (ZnO@CS) nanocomposite-based solid-phase extraction (SPE) cartridge was developed for atrazine removal. The ZnO@CS-based SPE cartridge achieved 98% recovery of atrazine under optimized conditions.

## Graphical abstract

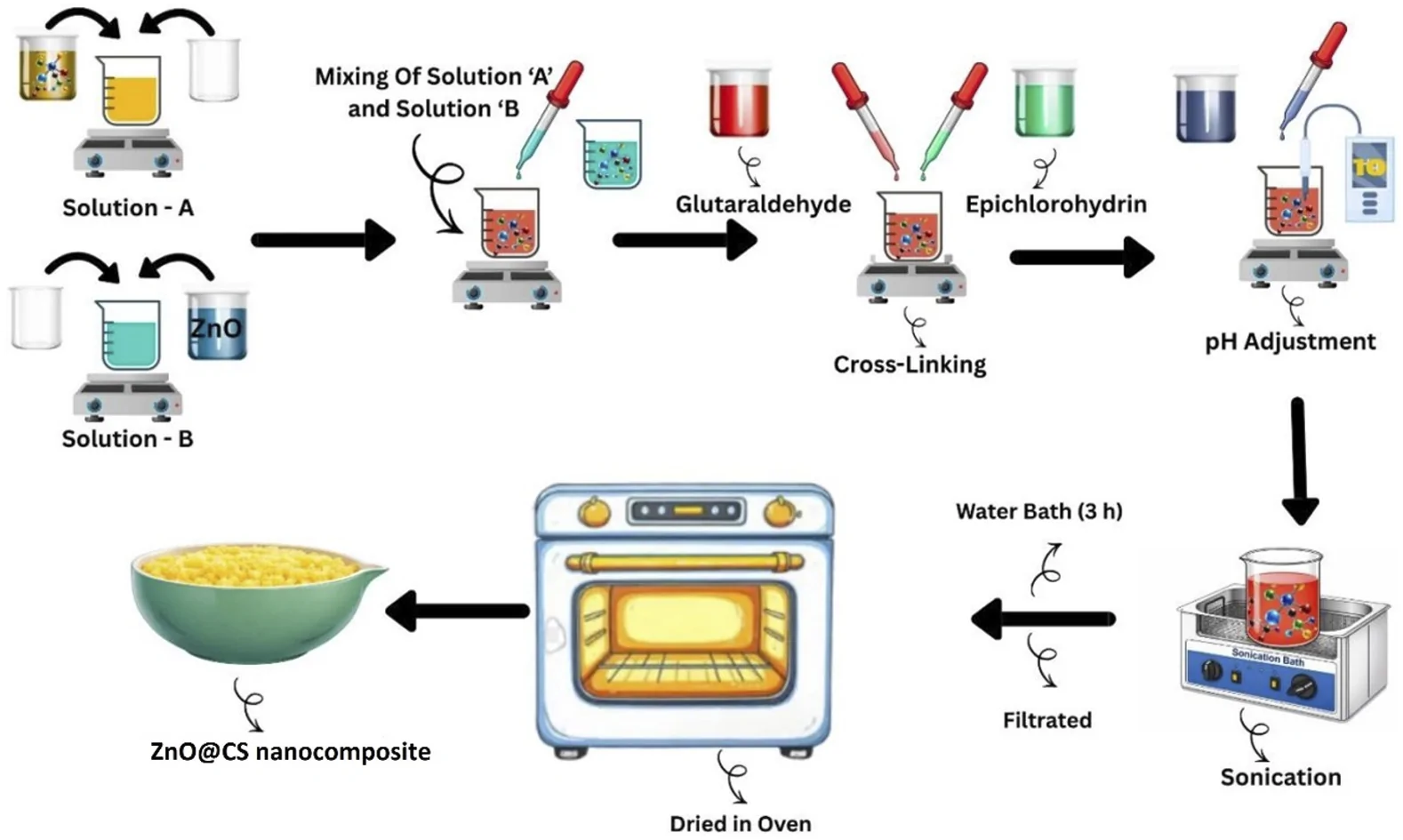

## 1. Introduction

Pesticides are persistent organic pollutants applied in agricultural fields that contaminate freshwater bodies, such as lakes, canals, rivers, and groundwater.^1–4^ Atrazine, chemically called 2-chloro-4-ethylamino-6-isopropylamino-1,3,5-triazine, is a widely used herbicide in various agricultural areas, which significantly contaminates water resources, such as rivers, canals, lakes, and groundwater, through surface runoff and leaching.^5–8^ The use of atrazine has been increasing every year both in developed and developing nations. Research showed that atrazine is primarily applied to agricultural crops, including sugarcane, sorghum, and corn, in the United States, with an estimated annual usage of 70 to 80 million pounds (32 000 to 36 000 metric tons).^9^ In Pakistan, atrazine is also used at almost 2 kg per hectare in maize crops.^10,11^ Atrazine can be found in the environment at considerable concentrations of up to 224 parts per billion in streams, 42 parts per billion in surface waters, 102 parts per billion in farmland water basins, and 21 parts per billion in groundwater. It has been declared a restricted-use pesticide by the U.S. Environmental Protection Agency (EPA) due to its environmental persistence and potential health risks. In addition, the EPA has provided guidelines and set a maximum contaminant level (MCL) of 3 ppm in drinking water under the Safe Drinking Water Act. The main purpose of this regulation is to improve living standards by safeguarding individuals from long-term exposure.

To reduce interference and increase analyte concentration for precise measurements, extracting pesticides before water sample analysis is essential. To date, various extraction techniques, such as liquid–liquid extraction (LLE), SPE, liquid-phase microextraction (LPME), stir-bar sorptive extraction (SBSE), magnetic SPE (MSPE), dispersive liquid–liquid microextraction (DLLME), and solid-phase microextraction (SPME), have been successfully applied for the extraction of various pesticides, with several advantages.^12–17^ Among these extraction methods, SPE is one of the most widely used and reliable methods for extraction and detection of various pesticides because of its excellent recovery rate and compatibility with various detection equipment.^18^ SPE is a highly effective and cost-effective technique for the extraction and detection of pesticides due to its high recovery rate and compatibility with various detection instruments. The adsorption process within the SPE matrix relies on the adsorption affinities between pesticides and the sorbent. In physical adsorption, weak forces like van der Waals attractions govern the interaction, while chemical adsorption involves the formation or breaking of chemical bonds, leading to stronger affinities between the pesticide and the SPE bed. The overall extraction efficiency depends on both covalent and non-covalent interactions between adsorbates (*e.g.*, pesticides) and the sorbent material. Covalent interactions can result in irreversible adsorption, rendering the sorbent unusable for future cycles. Conversely, non-covalent interactions, such as hydrogen bonding, van der Waals forces, hydrophobic interactions, and electrostatic attractions, are reversible and allow for efficient pesticide determination.^19^ To date, various functional SPE cartridge stationary phase media, including silica,^20,21^ inorganic frameworks,^22,23^ covalent organic frameworks,^24,25^ metal–organic frameworks,^26–28^ graphene^29–31^ and other carbon-based materials,^32,33^ have been developed to improve their extraction properties. Wang *et al.*^4^ developed a phosphoester-functionalized covalent organic framework anchored onto magnetite nanoparticles (Fe_3_O_4_@P-ZCOF) for the rapid, simultaneous extraction of 14 hydrophilic and hydrophobic organophosphate pesticides from environmental water, followed by UHPLC-MS/MS determination. The system was used to successfully extract 14 organophosphates within 6 minutes. The limits of detection (LOD, S/N = 3) for wastewater, reservoir water and tap water ranged from 0.01 to 1.50 ng L^−1^, 0.01 to 1.00 ng L^−1^ and 0.01 to 2.00 ng L^−1^, respectively. Moreover, molecularly imprinted polymer (MIP)-based sorbents have emerged as highly selective alternatives for these applications. Lang *et al.*^17^ reported a membrane-protected multi-template MIP (mt-MIP) micro-SPE device using mesoporous silica-modified multiwalled carbon nanotubes (MWCNTs@mSi) as a supporting scaffold. They successfully extracted and determined five chlorophenols (CPs) in leather matrices, followed by quantification with high-performance liquid chromatography (HPLC). They established that the mt-MIPs-µ-SPE-HPLC method demonstrated satisfactory linearity of 2.0–200 µg L^−1^, high sensitivity, with LODs of 0.17–0.52 µg L^−1^, and a high enrichment factor (70.6–144). In addition, carbon-based sorbents, including natural carbon materials, biochar, graphene, activated carbon, CNTs, fullerene, carbon dots, carbon fiber, doped-carbon materials, and carbon composites,^34–38^ have been widely used as a highly efficient sorbent materials inside SPE cartridges due to their exceptional physicochemical properties, including large specific surface areas, high porosity, tunable surface chemistry, and strong π–π interactions or hydrophobic effects that facilitate efficient pesticide retention. Such diverse carbon architectures offer advantages in terms of adsorption capacity, selectivity, reusability, and compatibility with green analytical protocols, making them promising alternatives or complements to traditional commercial sorbents (*e.g.*, C18 and Oasis HLB) for trace-level pesticide monitoring of environmental waters.^19^

Besides these materials, chitosan-based metal oxide nanocomposites have emerged as promising SPE sorbents for the extraction and recovery of different pesticides and other micropollutants from water.^39,40^ Chitosan, a naturally abundant cationic polysaccharide, possesses highly reactive functional groups, primarily hydroxyl (–OH) and amino (–NH_2_) moieties, that serve as key coordination and binding sites for organic pollutants.^41^ These groups enable effective capture of various pesticide classes, including organophosphates and organochlorines, through mechanisms such as hydrogen bonding, electrostatic interactions, and chelation. The intercalation of inorganic metal oxide NPs, such as zinc oxide (ZnO),^42^ into the chitosan matrix significantly enhances the composite's physicochemical properties, including surface area, stability, and adsorption affinity. Moradi Dehaghi *et al.*^43^ developed a chitosan–ZnO NP (CS–ZnONP)-based SPE cartridge, in which the CS–ZnONPs served as a stationary phase, and used it for permethrin pesticide extraction. They achieved near-complete removal (99%) of permethrin from an aqueous solution (25 mL, 0.1 mg L^−1^) at ambient temperature and neutral pH using 0.5 g of sorbent. Similarly, a copper-coated chitosan nanocomposite (CuCH) has also been reported for the removal of malathion from agricultural runoff, with good recovery.^44^ Moreover, Badawy *et al.* demonstrated chitosan–CuO NP-based SPE cartridges and achieved efficient removal of multiple pesticides, including parathion and methyl parathion, from water samples under optimized conditions (pH 7, 25 °C, 25 min agitation).^12^ Although considerable research has been conducted on chitosan-based adsorbents for the effective removal of pesticides and related molecules, several challenges remain unresolved. In particular, the relatively poor mechanical integrity, limited chemical stability, and insufficient reusability of pristine or minimally modified chitosan materials can restrict their practical application in complex environmental matrices. Therefore, a smart chemical strategy is required to enhance the physicochemical properties and adsorption performance of chitosan-based materials, thereby enabling their efficient and sustainable application in pesticide removal.

In this work, a novel ZnO@CS nanocomposite was synthesized *via* a sol–gel method and employed as an innovative sorbent material in an SPE cartridge for the extraction of atrazine from real water samples for the first time. The as-prepared nanocomposite was packed into syringe cartridges to serve as the stationary phase for efficient pesticide retention. The extraction performance of the fabricated SPE cartridges was systematically evaluated across five different atrazine concentrations (in µg L^−1^), prepared from an atrazine standard. Further, key operational parameters influencing adsorption performance, such as sample pH, agitation (contact) time, temperature, and sample volume, were initially screened and optimized using UV-Vis spectrophotometry to monitor residual atrazine levels in the effluent. Then, precise quantification of atrazine concentrations before and after extraction was performed using LC-MS/MS.

## 2. Experimental work

### 2.1. Materials

Atrazine (99.7%, with a molecular weight of 215.68) was purchased from Shanghai Macklin Biochemical Co., Ltd, China. Chitosan (3.60 × 10^5^ Da and 88% degree of deacetylation), glutaraldehyde (50%), epichlorohydrin (99%), zinc oxide (ZnO) (99%), acetic acid (AR grade), nitric acid, sodium hydroxide (1 N), HPLC-grade acetonitrile, and methanol were purchased from Sigma-Aldrich, Karachi, Pakistan.

### 2.2. Instrumental methods and characterization techniques

An FTIR spectrometer (PerkinElmer 1600, USA) with a LiTaO_3_ detector was used to confirm the chemical bonding and functionality of the nanocomposite. The zeta potential was measured using a Malvern Zeta-Nano-sizer (Nano-ZS L9002296 Scrouge). The surface morphology of the materials was observed by scanning electron microscopy (SEM) using a JEOL JSM-5300 microscope (JEOL Ltd, Japan). X-ray diffraction (XRD) patterns were collected using an X-ray diffractometer (Bruker D8 Advance, Germany). A Lambda 365 UV-Vis spectrometer (PerkinElmer, USA) was used. Finally, an HD 2070 ultrasonic homogenizer with HF was used as a generator. Additionally, a hot plate with a magnetic stirrer (IKA-Werke GmbH & Co., Breisgau-Hochschwarzwald, Germany), an oven (Heraeus Co., KG-Hanau, Germany), and electric balances (three- and four-digit readout, BL-410SLCD, Setra Systems Inc., 59 Swanson Rd., Boxborough, MA 01719, USA) were used for the characterization of materials and application studies of the fabricated SPE cartridge.

### 2.3. Synthesis of ZnO@CS nanocomposite

The ZnO@CS nanocomposite was prepared using a sol–gel method, as shown in Scheme 1. In the first step, solution A (chitosan polymer solution) was prepared by dissolving 4 g of chitosan in 100 mL of 1% (v/v) aqueous acetic acid with the help of a magnetic stirrer for 2 h (40 rpm). Subsequently, solution B (ZnO solution) was also prepared by dissolving 8 g of ZnO powder (99%) in 10 mL of concentrated nitric acid. After that, solution B was slowly added to solution A using a syringe under continuous magnetic stirring for 2 h until a homogeneous chitosan–ZnO polymer solution was obtained, as shown in Scheme 1.

**Scheme 1:**
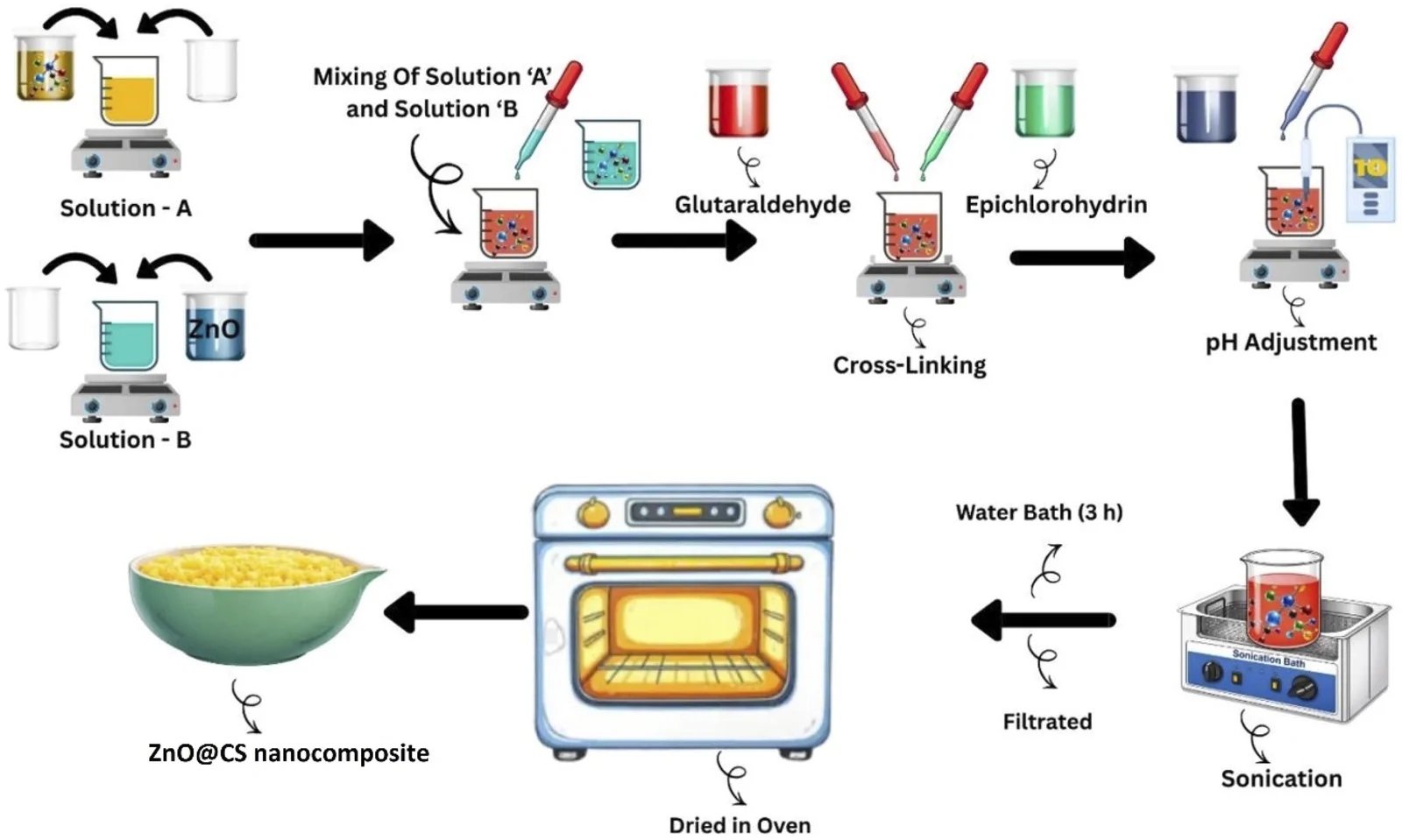
Schematic of the synthesis of the ZnO@CS nanocomposite by the sol–gel method involving multiple preparation steps.

In the second step, 12 mL of glutaraldehyde (50% v/v) was added to the chitosan–ZnO polymer solution dropwise under vigorous stirring to facilitate cross-linking. Then, the hard, spherical NPs were formed by cross-linking of glutaraldehyde to the amino (–NH) groups of chitosan. Subsequently, 8 mL epichlorohydrin (99%) was added to the reaction mixture with continuous stirring (Scheme 1). Epichlorohydrin further enhances the hardness of NPs by reducing the hydrophilicity of chitosan and completely binds the chitosan polymer with ZnO. The pH of the reaction solution was adjusted to 10 by adding a 1 N solution of NaOH with continuous stirring. The reaction mixture was then sonicated for 15 minutes using an ultrasonic sonicator (10 kHz speed and pulses or cycles at 9 cycles per second). Subsequently, the ZnO@CS cross-linked suspension was maintained in a water bath at 60 °C for 3 h until precipitates formed. The precipitate of the ZnO@CS nanocomposite was then collected by vacuum filtration using Whatman filter paper and washed thoroughly with DI water and dried in an oven at 70 °C for 3 h to remove residual moisture. Finally, a dark-yellow ZnO@CS nanocomposite was obtained and used as a cartridge sorbent for pesticide extraction (Scheme 1).

### 2.4. Atrazine standard stock solution preparation

A stock solution of atrazine (1000 ppm) was prepared by dissolving 25 mg of atrazine standard (MW: 215.68 g mol^−1^; 99.7% purity) in 25 mL of a 1 : 1 (v/v) mixture of methyl alcohol and DI water. Then, 10 ppm and 1 ppm solutions were further prepared from the stock solution. Subsequently, 100, 250, 500, 750, and 1000 ppb (µg L^−1^) atrazine solutions were prepared from a 1 ppm solution of atrazine for SPE cartridge loading. All standard solutions and dilutions were stored at −20 °C and used within four weeks to ensure chemical stability.^45^

### 2.5. Fabrication of the ZnO@CS-loaded SPE cartridge for atrazine recovery

Five standard atrazine concentrations (100, 250, 500, 750, and 1000 ppm) were prepared for the adsorption studies and SPE experiment for atrazine extraction, as shown in Scheme 2. Subsequently, the SPE cartridge was prepared by cutting 0.22 µm Whatman filter paper into a circular shape and fitting it at the bottom of a syringe to ensure proper placement. Then, 250 mg of as-prepared ZnO@CS nanocomposite was uniformly placed onto the filter paper and covered with another Whatman filter paper (0.22 µm) to securely immobilize the sorbent and minimize channeling. The assembled SPE cartridge was then used for the extraction and pre-concentration of atrazine from both synthetic and real water samples.

**Scheme 2:**
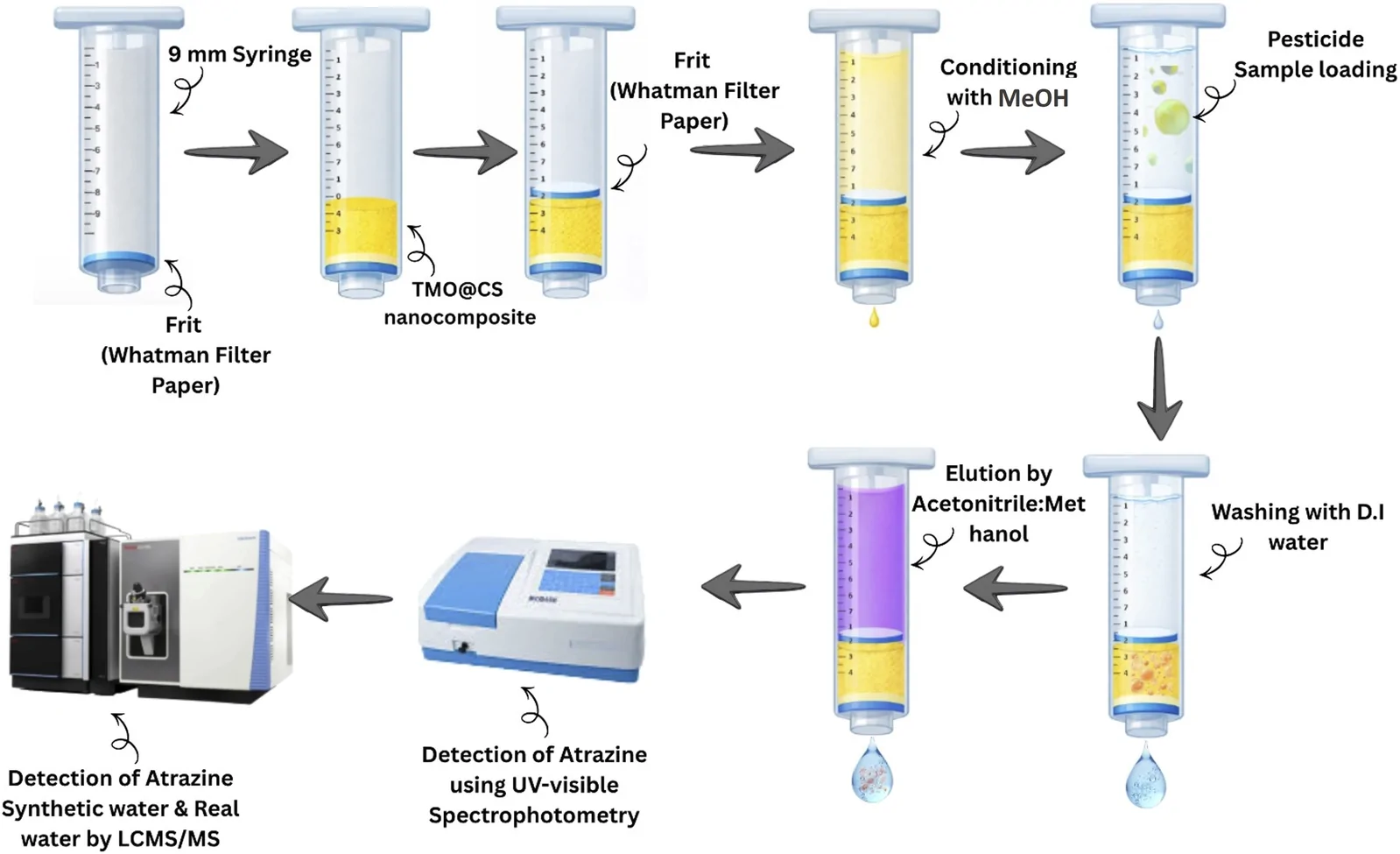
Schematic of the fabrication of the ZnO@CS nanocomposite and its loading into a SPE syringe cartridge for the selective extraction of the atrazine pesticide from water.

### 2.6. Optimization of parameters for the ZnO@CS-loaded SPE cartridge

In this study, various experimental parameters, such as concentration dosage, sorbent dosage, sample pH, and temperature, were optimized to achieve maximum atrazine extraction. Initially, the extraction performance of the as-prepared ZnO@CS-loaded SPE cartridge was evaluated using five different concentrations (1000, 750, 500, 250, and 100 ppm) of atrazine solution with a 250 mg dose of sorbent. After that, we also evaluated the extraction efficiency using different doses, such as 100, 200, 250, 300, and 400 mg of ZnO@CS nanocomposite. Among them, a dosage with excellent extraction efficiency was selected for further studies. The effect of sample pH on atrazine extraction was investigated using different pH values such as 5, 7, 9, and 10. The temperature effect was also examined by studying the extraction at different temperatures, such as 25 °C, 35 °C, 45 °C, and 55 °C. To obtain the best results, the cartridge was conditioned by passing 5 mL of methyl alcohol, followed by 5 mL of DI water. After adsorption, the retained pesticide was eluted using 5 mL of acetonitrile–methyl alcohol solution (1 : 1, v/v). The obtained eluate was measured with a UV-Vis spectrophotometer for quantification of the recovery.

Extraction recovery (ER, %) was calculated as the percentage of the initial analyte amount transferred into the eluent (eqn (1)) as follows:1
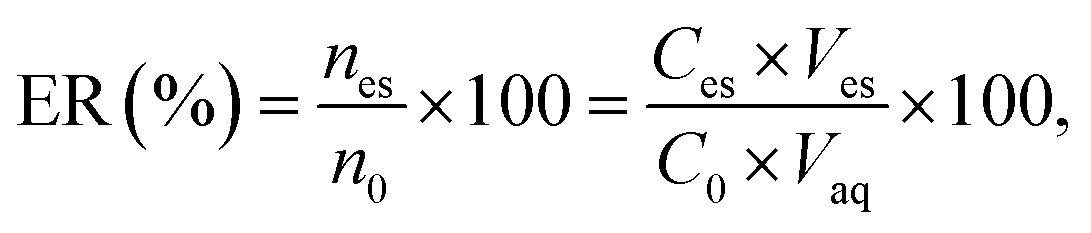
where *n*_0_ and *n*_es_ represent the initial and eluted amounts of atrazine, respectively, *C*_0_ and *C*_es_ are the initial and eluted atrazine concentrations, respectively, and *V*_aq_ and *V*_es_ denote the volumes of the aqueous sample and eluent, respectively.^46^

### 2.7. Kinetics studies

The adsorption rate onto the particle surface requires an extensive understanding of the adsorption process's kinetics, since these models may be used to illustrate how different factors affect the process's rate of adsorption. Furthermore, the mechanism of pesticide adsorption onto the adsorbent ZnO@CS nanocomposite is determined by adsorption kinetics. Pseudo-first-order and pseudo-second-order kinetic models were used to model the atrazine pesticide's kinetic data.

#### 2.7.1 Pseudo-first-order (PFO) kinetic model

Lagergren proposed the initial proposal for the PFO concept in 1898.^47^Eqn (2) describes the differential form of the PFO model as follows:

2 d*q*_*t*_/d*t* = *k*_1_(*q*_e_ − *q*_*t*_).

when *q*_0_ = 0 is met, integrating eqn (2) yields:

3 *q*_*t*_ = (1 − *e* − *k*_1_).

The PFO model is shown in its linearized form as follows (eqn (4)):

4 Ln(*q*_e_ − *q*_*t*_) = ln *q*_e_ − *k*_1_*t*.

The equilibrium adsorption quantity that the PFO model estimates is the PFO parameter, or *q*_e_. Silva *et al.*^48^ suggested that when the adsorption isotherm could be described by the linear model (eqn (5)), the PFO model was theoretically compatible and equivalent to the linear driving force (LDF) model.

5 *q*_e_ = *KC*_e_

The rate at which the adsorption equilibrium is reached is commonly expressed by the PFO parameter *k*_1_, but as eqn (1) demonstrates, *k*_1_ and (*q*_e_ − *q*_*t*_) are linked to the adsorption rate d*q*_*t*_/d*t*. When adsorption is slow, small *k*_1_ and large values of (*q*_e_ − *q*_*t*_) may be achieved. Therefore, using eqn (6) to compute the PFO rate is more accurate than comparing the *k*_1_ values to describe the adsorption rate:^49^

6 PFO rate = *k*_1_(*q*_e_ − *q*_*t*_)

#### 2.7.2 Pseudo-second-order (PSO) kinetic model

Initially, the adsorption of lead onto peat was modeled using the PSO model (eqn (7)). Afterward, the PSO model was extensively used to describe the adsorption processes. The PSO model has been used in most published studies to compute the adsorption rate constants and forecast the adsorption experimental data.

7 d*q*_*t*_/d*t* = *k*_2_ (*q*_e_ −)^2^

The integrated PSO model is described as follows (eqn (8)):

8 *q*_*t*_ = *q*_e_^2^*k*_2_*t*/1 + *q*_e_*k*_2_*t*.

The nonlinear PSO model is always converted into the linear form (eqn (9)) to determine the model parameters as follows:^49^

9 *t*/*q*_*t*_ = 1/*k*_2_ × 1 × *q*_e_^2^ + *t*/*q*_e_.

### 2.8. Adsorption isotherm studies

#### 2.8.1 Langmuir isotherm

The eqn (10) represents the Langmuir isotherm in its generalized form:

10 *C*_e_*q*_e_ = 1/*q*_m_*k*_l_ + *C*_e_*q*_m_

where (mg L^−1^) is the Langmuir adsorption constant, mg g^−1^ is the monolayer capacity of the adsorbent, mg g^−1^ is the equilibrium metal ion concentration on the adsorbent, and mg L^−1^ is the equilibrium atrazine concentration in solution.

The dimensionless constant known as the equilibrium parameter, or *R*_L_, from the following equation demonstrates the fundamental property of the Langmuir isotherm (eqn (11)):

11 *R*_L_ = 1/1 + *K*_l_*c*_o_

where (mg L^−1^) is the lowest starting concentration and (L mg^−1^) is the Langmuir constant associated with the adsorption energy.

The *R*_L_ value may suggest that the isotherm's form is irreversible (*R*_L_ = 0), favorable (0 < *R*_L_ < 1), linear (*R*_L_ = 1), or unfavorable (*R*_L_ > 1). The following equation defines the equilibrium parameter *R*_L_, a dimensionless constant expressing the Langmuir isotherm's fundamental properties:

12 *R*_L_ = (1/1 +).

*C*_o_ is the starting concentration, and *K*_L_ is the Langmuir adsorption constant. Using the formula above, one can compute the value of *R*_L_. The adsorption process can be classified as irreversible (*R*_L_ = 0), advantageous (0 < *R*_L_ < 1), linear (*R*_L_ = 1), or unfavorable (*R*_L_ > 1).

#### 2.8.2 Freundlich isotherm model

The Freundlich isotherm model describes heterogeneous reversible adsorption on surfaces that are not limited to monolayer formation. The Freundlich isotherm model can be expressed by eqn (13) as follows:

13 Log *q*_e_ = log *K*_f_ + 1/*n* log *C*_e_.

The equilibrium atrazine concentration in the solution is expressed as mg L^−1^, and the equilibrium atrazine concentration on the adsorbent is m g^−1^. The Freundlich constants that may be linked to the adsorption intensity and capacity are (m mg^−1^), respectively.

### 2.9. Water sample collection

All samples were collected, handled, labelled and transported according to Standard Operating Procedures (SOPs) for water sampling. The sampling area was selected based on intensive sugarcane cultivation, where atrazine is commonly used. The real water samples were collected from the Tando Muhammad Khan District, Sindh, Pakistan, as shown in Fig. 1. In this area, agricultural runoff drains directly into the Phuleli Water Canal and subsequently mixes with the Akram canal, Pinyari, and Indus River canal systems. A total of six real water samples were collected, including two samples from sugarcane agricultural fields, two from groundwater sources, and two samples from irrigation watercourses from Sulaiman Goath and Mir Tokali villages (Khattar area). All samples were collected in clean, properly labeled, closed containers and transported directly to the research laboratory under controlled conditions for further analysis.

**Fig. 1:**
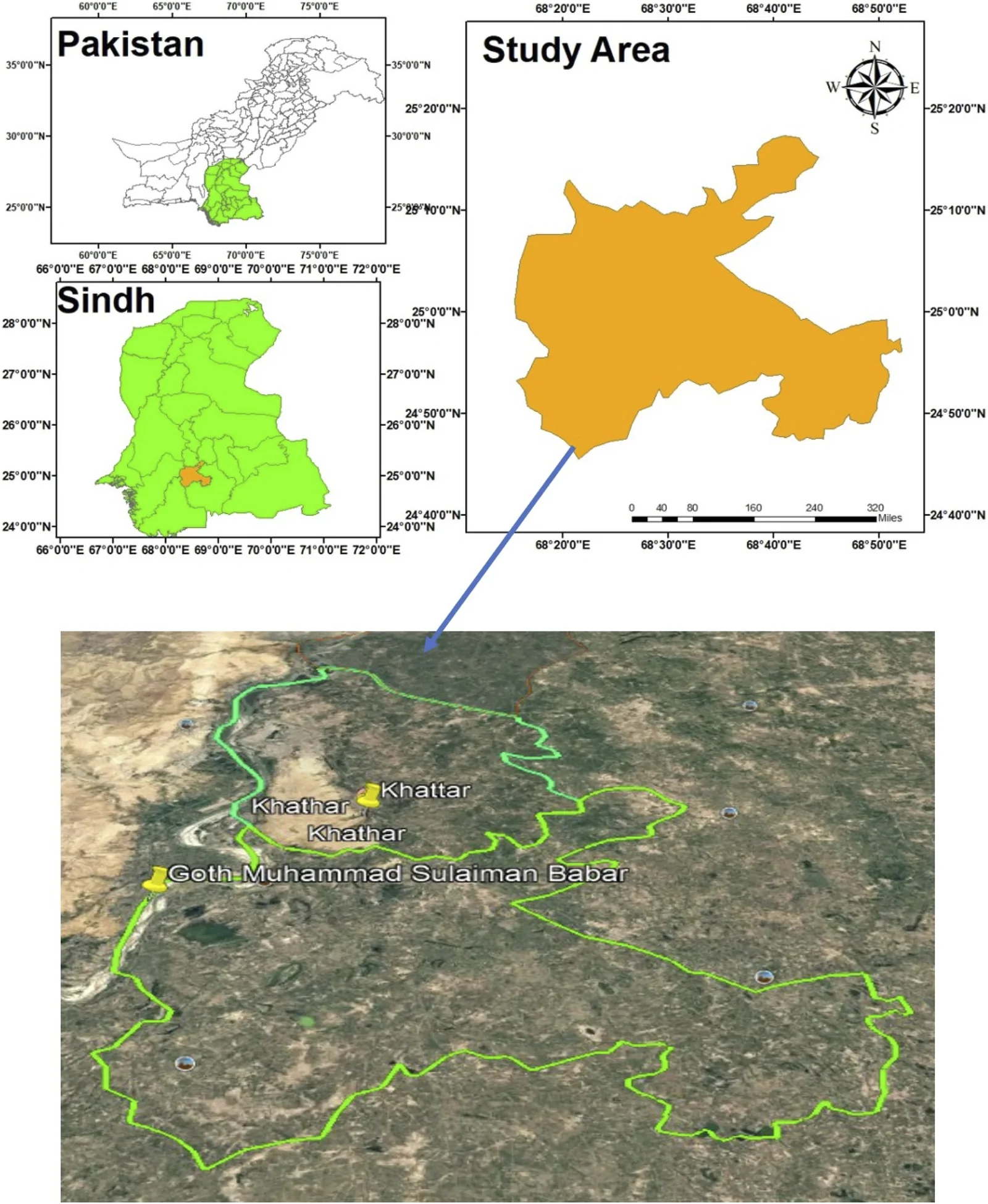
GIS map of the study area and location of the Tando Muhammad Khan District, Sindh, Pakistan, where the real water samples were collected.

#### 2.9.1 Sample transportation and preservation

Initially, the physical parameters, including pH, total dissolved solids (TDS), electrical conductivity (EC), *etc.*, were measured for the collected samples in the field before transportation. Then, samples were preserved at 10 °C in a portable cooler using ice pads and transported to the laboratory for detection of atrazine concentrations following all SOPs. The real water sample was then filtered through a filtration assembly using 0.45 µm Whatman filter paper to remove the turbidity and suspended solids from the water, which can cause interference during extraction from the cartridge. After that, the pH of the samples was adjusted to pH 5 using 1 N hydrosulfuric acid dropwise until the pH reached 5, which is the best preservation pH for organochlorine pesticides in water. The samples were stored in amber glass bottles (dark color) and kept at 4 °C in a refrigerator for up to 40 days for further testing.

#### 2.9.2 Atrazine extraction from real water samples using ZnO@CS-loaded SPE cartridge

The as-prepared ZnO@CS-loaded SPE cartridge was employed for the extraction of atrazine from real water samples. First, the cartridge was conditioned by passing 5 mL of methyl alcohol through the stationary phase to activate the functionality of the sorbent materials. After that, the real sample (100 mL) was passed through a ZnO@CS cartridge at a controlled flow rate of 1 mL min^−1^. After loading the real sample, the cartridge was washed again with 5 mL of DI water to remove residual solvent and matrix interferences from the surface of the ZnO@CS sorbent. The loaded atrazine was then eluted using 5 mL of acetonitrile/methyl alcohol mixture (1 : 1). The collected eluate was filtered through a 0.22 µm syringe filter and stored at −20 °C prior to quantitative analysis using LC-MS/MS.

## 3. Results and discussion

### 3.1. Physicochemical characterization of the ZnO@CS nanocomposite

The as-synthesized ZnO@CS nanocomposites were characterized to evaluate their structural, morphological, and chemical properties prior to application. The surface morphology and particle size of the ZnO@CS nanocomposite were examined using SEM (Fig. 2a and b). The SEM images obtained at different magnifications showed particles mainly in the range of approximately 30–61 nm, including observed sizes of 30, 32, 50, 51, 57, 58, 60, and 61 nm at 100 00× magnification and a 1 µm scale. The nanoparticles exhibited irregular granular and spherical morphologies.

**Fig. 2:**
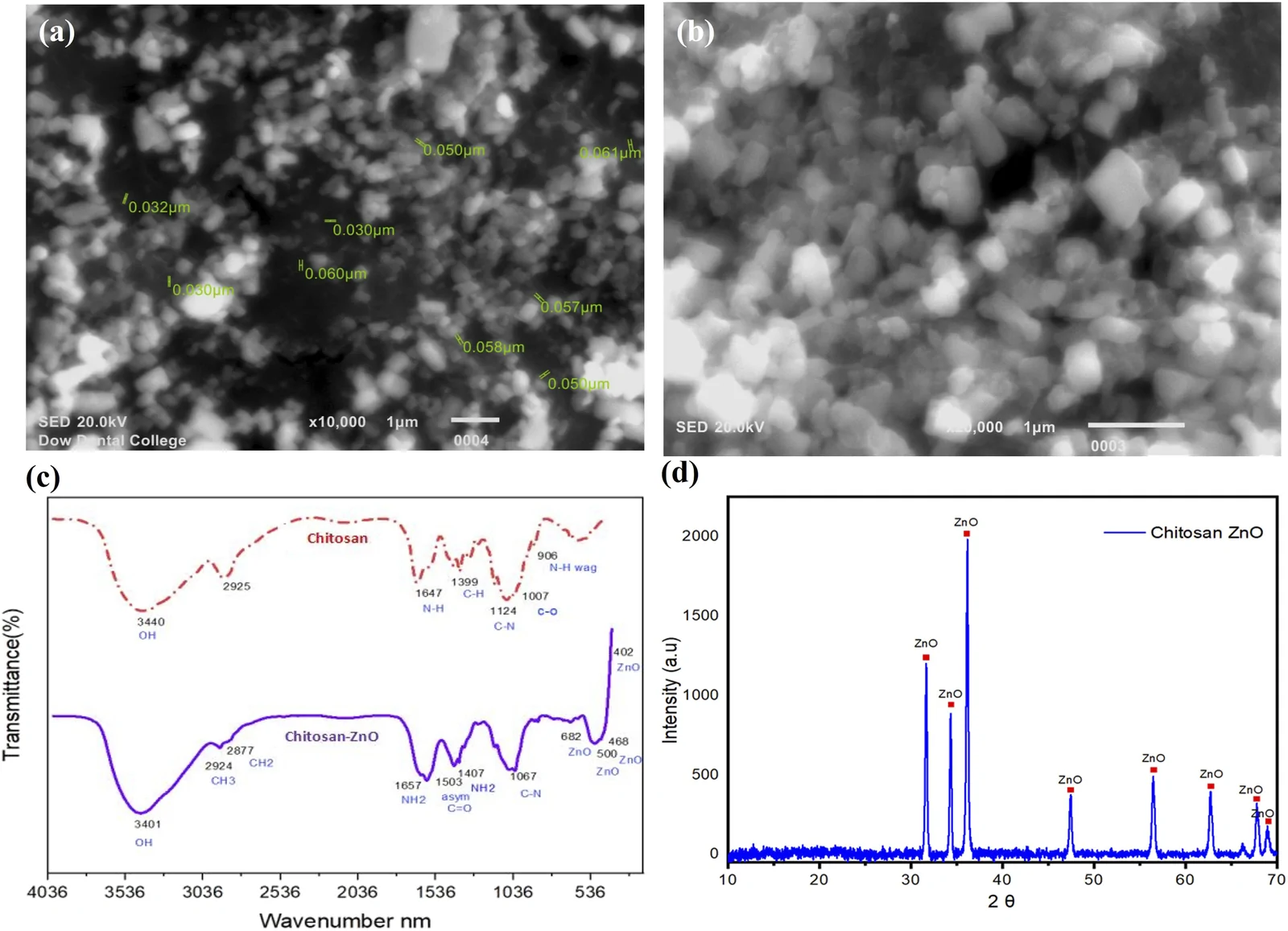
(a and b) Surface SEM images, (c) FTIR spectra, and (d) XRD pattern of the ZnO@CS nanocomposite.

Further, the elemental composition of the as-prepared ZnO@CS nanocomposite was conf3irmed by energy-dispersive spectroscopy (EDS), as shown in Fig. S1. The EDS analysis at 18.00 kV voltage and 3000× magnification showed that carbon (17.48%), zinc (21.61%), and oxygen (60.90%) are present. Zinc and oxygen are supplied by the ZnO phase in the composite, whereas the main source of carbon is the chitosan molecules.

FTIR spectroscopy was performed to identify the functional groups of the ZnO@CS nanocomposites and to confirm the interaction between ZnO and the chitosan matrix (Fig. 2c). A broad and intense absorption band centered at 3401 cm^−1^ corresponds to the overlapping stretching vibrations of hydroxyl (–OH) and amine (–NH_2_) groups of chitosan. The broadening of this peak indicates strong hydrogen bonding and possible coordination interactions between Zn^2+^ ions and the functional groups of chitosan. This study confirms that the ZnO NPs are chemically associated with the polymer matrix rather than being physically mixed. The absorption bands at 2924 cm^−1^ and 2877 cm^−1^ are attributed to the asymmetric and symmetric stretching vibrations of –CH_2_ and –CH_3_ groups of the chitosan backbone. These bands confirm that the primary polymer structure remains intact after nanocomposite formation. A characteristic band at 1657 cm^−1^ corresponds to amide I (C=O stretching vibration), while the band at 1553 cm^−1^ is assigned to amide II (N–H bending vibration). These bands indicate the presence of residual acetyl groups and confirm the preservation of chitosan's chemical structure. The peak observed at 1407 cm^−1^ is associated with C–N stretching and –NH_2_ deformation, whereas the band at 1067 cm^−1^ corresponds to C–O stretching vibrations of the polysaccharide ring structure.

Notably, several new absorption bands appeared in the low-wavenumber region at 682 cm^−1^, 500 cm^−1^, 468 cm^−1^, and 402 cm^−1^, which were attributed to characteristic bands of Zn–O stretching vibrations. These bands provide evidence for Zn–O bonds and support the presence of ZnO associated with the chitosan matrix. The interaction is likely mediated through coordination between Zn^2+^ ions and the –NH_2_ and –OH functional groups of chitosan, resulting in an organic–inorganic hybrid nanocomposite. Overall, the FTIR results support the presence of both chitosan functional groups and Zn–O bonds and are consistent with interactions between the two components. However, FTIR spectroscopy does not by itself establish a core–shell architecture or quantify the surface area. The coexistence of amine and hydroxyl groups with metal oxide components supports the potential of the material for pesticide extraction and water-treatment applications.

An XRD pattern of the ZnO@CS nanocomposite was recorded to determine the crystalline structure, phase purity, and orientation of the as-synthesized nanocomposite (Fig. 2d). The diffraction peaks were observed at 2*θ* values of 31.80°, 34.35°, 35.42°, 36.21°, 46.34°, 47.27°, 56.50°, 62.70°, 66.24°, and 68.96°. These peaks correspond to the characteristic crystallographic planes of ZnO, confirming the formation of a hexagonal wurtzite crystalline structure. The prominent diffraction peaks at approximately 31.7°, 34.3°, 36.2°, 56.5°, and 62.7° are well indexed to the (100), (002), (101), (110), and (103) planes of ZnO, respectively, consistent with the standard (JCPDS No. 36-1451), confirming the formation of the crystalline ZnO phase within the composite. The sharp and intense nature of these peaks indicates good crystallinity of the synthesized NSs. Moreover, the observed diffraction pattern closely matches previously reported results,^39^ confirming the successful formation of crystalline ZnO within the chitosan matrix. Further, AbdElhady *et al.* also reported diffraction peaks at 2*θ* values of 31.80°, 34.50°, 36.60°, 47.40°, 56.60°, 62.70°, 66.40°, 68.10°, and 69.10° for ZnO–chitosan nanocomposites synthesized using a microwave-assisted heating method.^50^ These diffraction positions are in close agreement with those observed in the present study, particularly for the characteristic reflections of crystalline ZnO. The consistency between the reported and experimental diffraction patterns provides additional evidence for the successful incorporation of ZnO into the chitosan matrix and confirms the retention of its hexagonal wurtzite crystal structure. Furthermore, Madhan *et al.*^51^ reported comparable crystallographic characteristics for chitosan–ZnO NSs synthesized using plant-mediated methods. They reported diffraction peaks at 2*θ* = 32.95°, 35.27°, 45.17°, 50.36°, 58.82°, 60.08°, 61.27°, 64.07°, 70.29°, and 73.79° that were indexed to the (100), (101), (102), (110), (103), (200), (112), (201), (004), and (202) planes, respectively, and were closely associated with the hexagonal wurtzite structure of ZnO according to JCPDS No. 36-1451. Additionally, weak peaks observed at lower 2*θ* values (approximately 21°–28°) are attributed to the semi-crystalline nature of chitosan, indicating the presence of the organic polymer phase in the nanocomposite. These results confirm the successful formation of an organic–inorganic nanocomposite structure, where crystalline ZnO NPs are well incorporated within the chitosan matrix.

The formation of the ZnO@CS nanocomposite was also monitored using UV-Vis spectroscopy (Fig. S2). The UV-Vis peak of ZnO@CS was found in the wavelength region of 370 nm (Fig. S2). The results show the reliability and functional characteristics of the synthesized NPs for atrazine extraction from water. A previous study showed that the UV-Vis spectra of the as-prepared chitosan–ZnO NSs also exhibited a peak at 370 nm.^50,52^ Furthermore, in another study, the formation of a UV peak at 355 shows the characteristic features of ZnO only, and the formation of a 360 nm scanning peak is characteristic of chitosan and ZnO NPs without cross-linking.^52^ Moreover, Madhan *et al.*^51^ reported that the UV-Vis spectra of synthesized ZnO NPs and CS/ZnO nanocomposites were recorded at 370 nm using a Shimadzu UV-1800 spectrophotometer at 300 to 600 nm.

### 3.2. Zeta potential analysis

The surface charge value in zeta potential is a useful indicator of the electrostatic behavior and dispersion stability of colloidal particles. In this study, the zeta potential of the synthesized ZnO@CS nanocomposite was −12.6 mV, and the zeta deviation was 3.47 mV at 2 µm, 25 °C, 180.2 count rate (kbps), 0.8872 cP viscosity, 12 zeta runs, and 0.0434 mS cm^−1^ conductivity, showing acceptable measurement quality, measured using a Malvern zeta potential instrument, as shown in Fig. 3. The negative zeta-potential value indicates a net negative surface charge under the measurement conditions. However, zeta potential does not provide a direct measurement of the specific surface area and should not be interpreted as evidence of a large BET surface area. Quantitative surface-area characterization would require BET analysis.

**Fig. 3:**
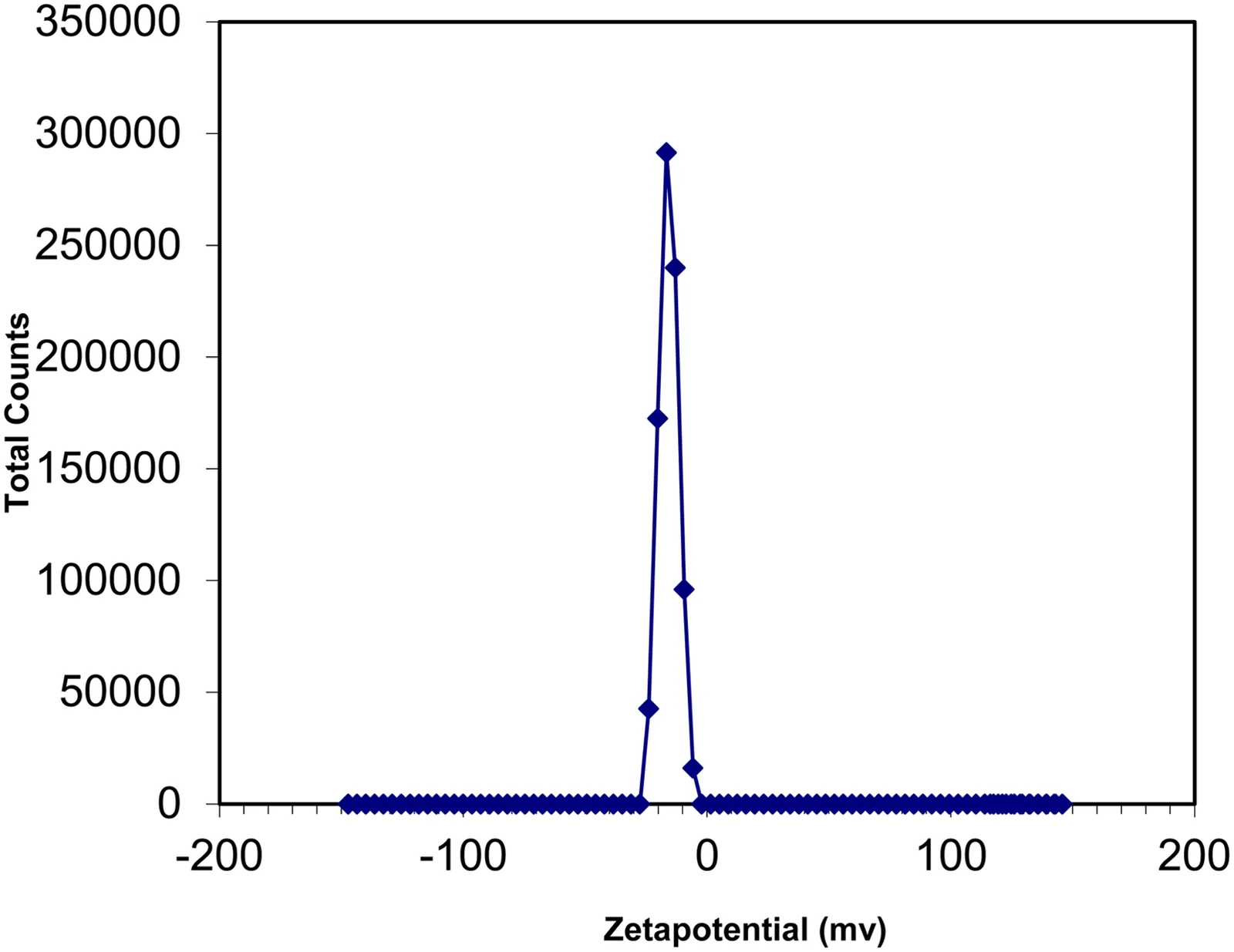
Surface charge value of the ZnO@CS nanocomposite analyzed by a zeta potential analyzer.

## 4. Extraction recovery of atrazine using a ZnO@CS-loaded SPE cartridge

The extraction recovery formula ER% = *n*_es_/*n*_0_ × 100 was used for a 250 mg dosage of synthesized ZnO@CS nanocomposite to extract atrazine pesticides at 1000 µg L^−1^, 750 µg L^−1^, 500 µg L^−1^, 250 µg L^−1^, and 100 µg L^−1^ (Fig. 4). The maximum extraction recovery was achieved at 100 µg L^−1^ concentration. The dosage research was carried out as optimum parameter study 1 at 100 µg L^−1^, and a second pH study was carried out to determine the impact of varying pH on the extraction recovery of the cartridge at 100 µg L^−1^. Additionally, the effects of various temperatures, as well as agitation duration and sample volume during elution at 100 ppb, were examined. The study was conducted with a loading of 250 mg of sorbent to determine the extraction and recovery of the ZnO–chitosan nanocomposite.^12^ The highest extraction rates (98%) were observed for lambda-cyhalothrin. The findings showed that while the adsorption or retention % increased as the temperature and pH increased, the ideal adsorption percentage was found at pH 7 and 25 °C for lambda-cyhalothrin with 250 mg of sorbent. Under the same conditions, other pesticides such as diazinon (60%), thiophanate methyl (94%), methomyl (41%), fenamiphos (95%), and abamectin (98%) showed lower extraction recoveries in comparison to lambda-cyhalothrin at 10 mg per L concentration.

**Fig. 4:**
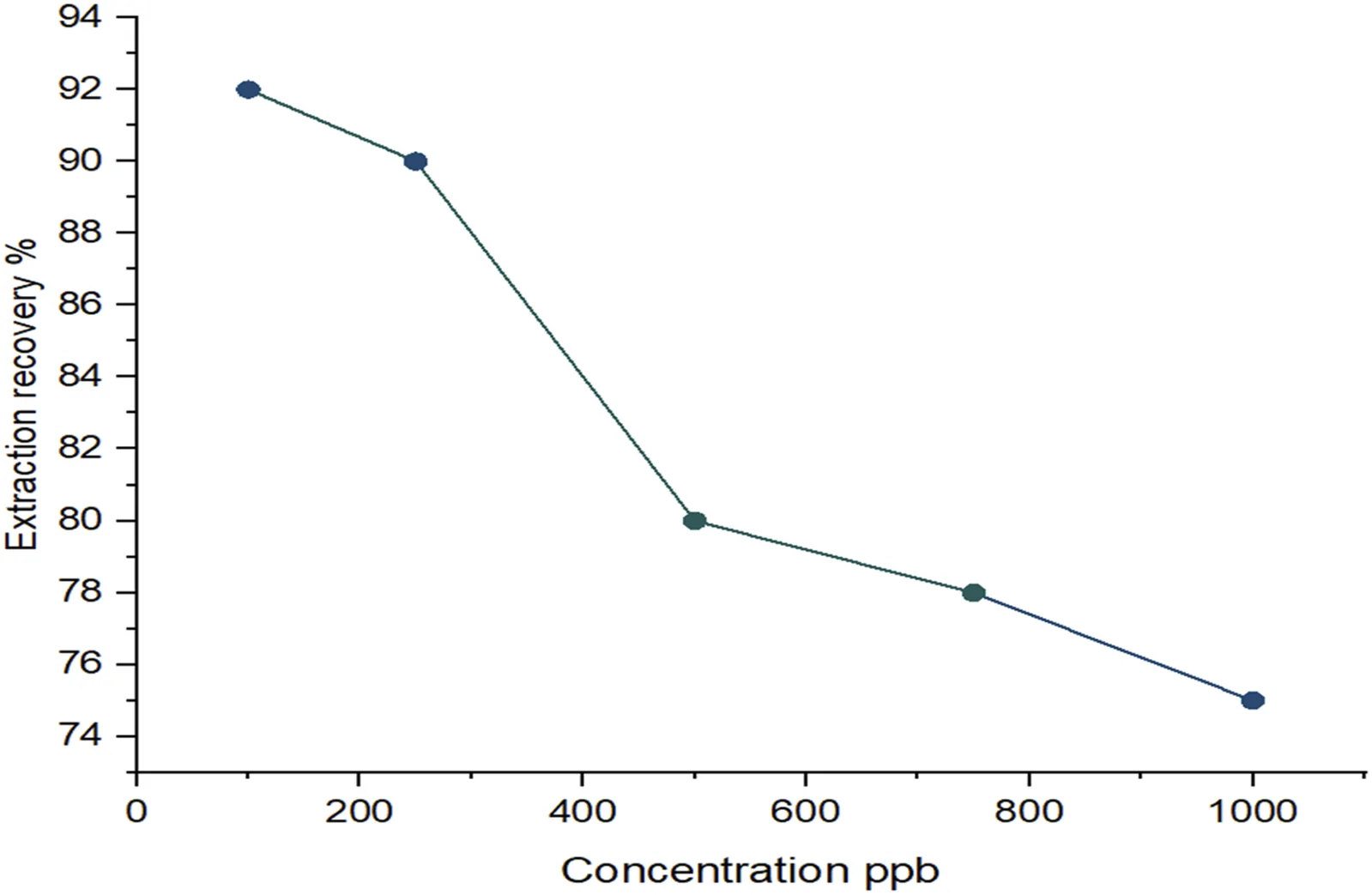
Extraction efficiency of the fabricated ZnO@CS nanocomposite cartridge at different atrazine concentrations (1 ppm, 100 ppb, 250 ppb, 500 ppb, 750 ppb, and 100 ppb) using a 250 mg dose of the ZnO@CS sorbent.

### 4.1. Optimization of adsorption parameters

#### 4.1.1 Effect of adsorbent dosage

A key factor that influences the adsorption capacity of a sorbent for a fixed initial sorbate concentration is the amount of adsorbent used. In the first experiment, separate syringe cartridges were prepared by loading different dosage amounts (in milligrams) of the ZnO@CS nanocomposite. The extraction procedure was carried out at a concentration of 100 µg L^−1^ (100 ppb). As shown in Fig. 5a, the extraction recovery reached 92% at a dosage of 250 mg for a 100 µg L^−1^ concentration. Therefore, to further optimize the system parameters such as dosage, pH, temperature, contact time, and sample volume, the concentration of 100 µg L^−1^ was selected to evaluate the extraction performance of the fabricated ZnO@CS nanocomposite cartridge. After complete dose optimization, the maximum recovery was observed at 300 mg for 100 µg L^−1^. This result indicates that increasing the adsorbent dosage increases the number of available active sites and enhances the overall surface area for adsorption. However, beyond a certain amount, the improvement becomes limited. This may be due to particle aggregation and increased sorbent–sorbent interactions, which can reduce the effective active surface area available for pesticide binding. Moradi Dehaghi *et al.*^43^ reported 0.5 g of chitosan–ZnO nanostructures (NSs) as the optimum adsorbent dosage for the removal of permethrin pesticide from aqueous solution. They suggested that at higher sorbent concentrations (>0.5 g), the availability of active sites may decrease. Although increasing the composite dose initially increases the total surface area, excessive sorbent concentration may result in stronger sorbent–sorbent interactions rather than sorbate–sorbent interactions, thereby reducing adsorption efficiency.

**Fig. 5:**
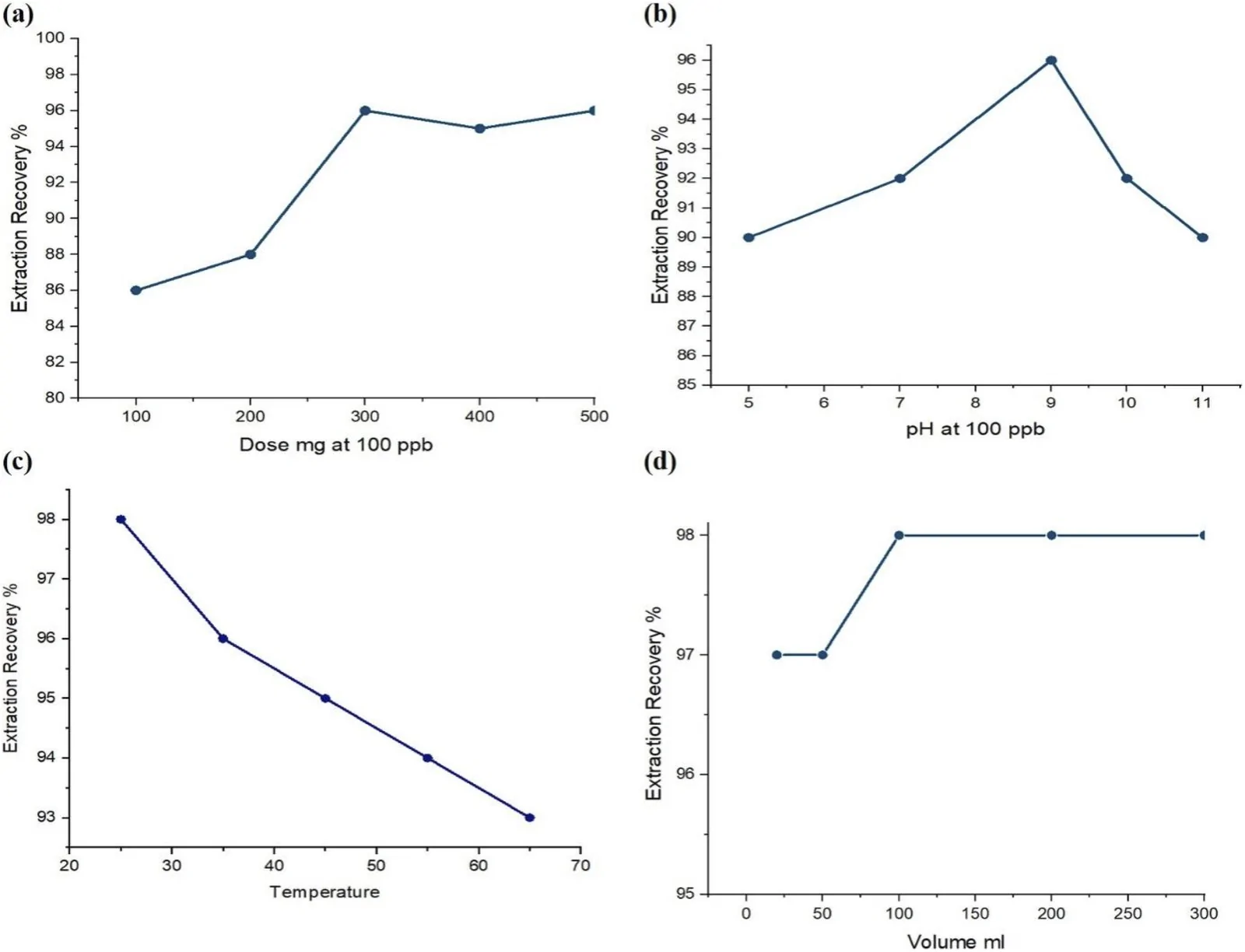
Optimization of the SPE conditions for atrazine extraction using the ZnO@CS-loaded cartridge: (a) effect of the ZnO@CS sorbent dosage on the extraction recovery at an atrazine concentration of 100 ppb, (b) effect of the solution pH on the extraction recovery using 300 mg of ZnO@CS at 100 ppb atrazine, (c) effect of the temperature on the extraction recovery using 300 mg of ZnO@CS at 100 ppb atrazine, and (d) effect of the sample volume on the extraction recovery using 300 mg of ZnO@CS at 100 ppb atrazine. Unless otherwise specified, the experiments were conducted at pH 9, 25 °C, and a contact time of 20 min.

#### 4.1.2 Effect of pH optimization

The effect of pH on the extraction efficiency of atrazine was studied under acidic, neutral, and alkaline conditions over the pH range of 5 to 11, as shown in Fig. 5b. The experimental conditions were optimized, and a 300 mg dose of ZnO@CS adsorbent was used, along with a constant concentration of atrazine of 100 ppb. First, atrazine solutions at pH 7.0 were used. The pH of the neutral solution was adjusted to alkaline and acidic using 1 N NaOH and 1 N H_2_SO_4_, respectively. After that, the extraction performance of the ZnO@CS-loaded SPE cartridge was tested at pH 5, 7, 9, 10, and 11 to find the optimal pH for atrazine extraction.

The pH value significantly affects the surface charge of the stationary phase inside the cartridge and the structural behavior of the sorbent.^43^ The amine (–NH_2_) and hydroxyl (–OH) groups present in chitosan act as potential binding sites. Depending on the pH, these functional groups may undergo protonation or deprotonation, which directly influences surface complexation and electrostatic interactions. As shown in Fig. 5b, the highest extraction recovery (above 95%) was achieved at pH 9. Therefore, pH 9 was selected as the optimum condition for maximum extraction efficiency. This may be due to stronger electrostatic interactions between the positively charged sorbent surface and negatively charged pesticide species under slightly basic conditions. In the pH range of 5–7, the sorbent surface may become highly protonated due to the presence of excess hydronium ions. This can influence the interaction between the sorbate and the functional groups of the sorbent. On the other hand, the decrease in adsorption capacity beyond pH 9 may be attributed to competition between hydroxyl ions and pesticide anions for the available active sites on the sorbent surface. A similar trend was also observed for the extraction rate of permethrin pesticide, which increased as the pH increased to 8 when using chitosan–ZnO NSs.^43^

#### 4.1.3 Temperature optimization

The effect of temperature on the extraction efficiency of atrazine was also investigated. Experiments were carried out at 25 °C, 35 °C, 45 °C, 55 °C, and 65 °C, using the optimized dose constant of 300 mg of ZnO@CS nanocomposite. As shown in Fig. 5c, the highest extraction recovery (E.R.%) of 97% was achieved at 25 °C, and no significant improvement was observed at higher temperatures. A similar temperature-dependent behavior was also reported for chitosan–ZnO NPs for the extraction of imidacloprid, thiophanate-methyl, fenamiphos, abamectin, diazinon, and λ-cyhalothrins in the literature.^12^ In that work, optimum extraction performance was also achieved at 25 °C. This study suggests that room-temperature conditions are sufficient for maximum adsorption efficiency, and increasing temperature does not provide additional benefits.

#### 4.1.4 Sample volume optimization

The effect of sample volume on the extraction efficiency of atrazine was evaluated by passing different volumes of atrazine standard solutions (20, 50, 100, 200, and 400 mL) through the ZnO@CS-loaded SPE cartridge using the same dosage of sorbent (300 mg). This study aimed to evaluate the efficiency of the fabricated cartridge compared to commercial C-18 cartridges, which typically handle up to 500 mL of sample (Fig. 5d). Tümay Özer *et al.*^53^ investigated the extraction of organophosphorus pesticide from polymeric bead-based sorbents and reported that 200 mL was the optimal sample volume for the SPE cartridge, providing effective retention and subsequent elution of the target analytes.

#### 4.1.5 Time optimization

The influence of contact time on the extraction of atrazine was also studied at 20, 40, 60, 80, and 100 min using 100 mL of atrazine solution (100 ppb). The experiments were carried out using 300 mg of ZnO@CS nanocomposite at pH 9 and 25 °C. Fig. 6 shows that the extraction recovery increased progressively with increasing contact time, from approximately 95% at 20 min to 98% at 100 min. The most significant increase in recovery was observed between 20 and 40 min; however, approximately 96% recovery was also observed between 40 and 60 min. Further, we also observed that recovery subsequently increased to 97% at 80 minutes, and the maximum (98%) recovery was achieved at 100 minutes. This increase in extraction recovery efficiency is possible due to the presence of various absorption sites on the surface of the sorbent (ZnO@CS), which facilitates rapid interaction with the atrazine molecules during the early stages of the extraction process. As the contact time increased, these sites were gradually occupied, resulting in a slower increase in extraction efficiency. However, the slow improvement observed between 60 and 100 minutes suggests that the adsorption process had not fully reached equilibrium within the timeframe of the investigation. Because it produced the best extraction recovery of almost 98%, 100 minutes was determined to be the ideal contact period under the testing conditions. This study is supported by literature;^43^ Moradi Dehaghi *et al.*^43^ reported that adsorption initially occurs rapidly due to abundant active surface sites but slows down as these sites become occupied, eventually reaching equilibrium. In their study, pesticide sorption increased from 78% to 99% as contact time increased from 10 to 90 min, after which it stabilized.

**Fig. 6:**
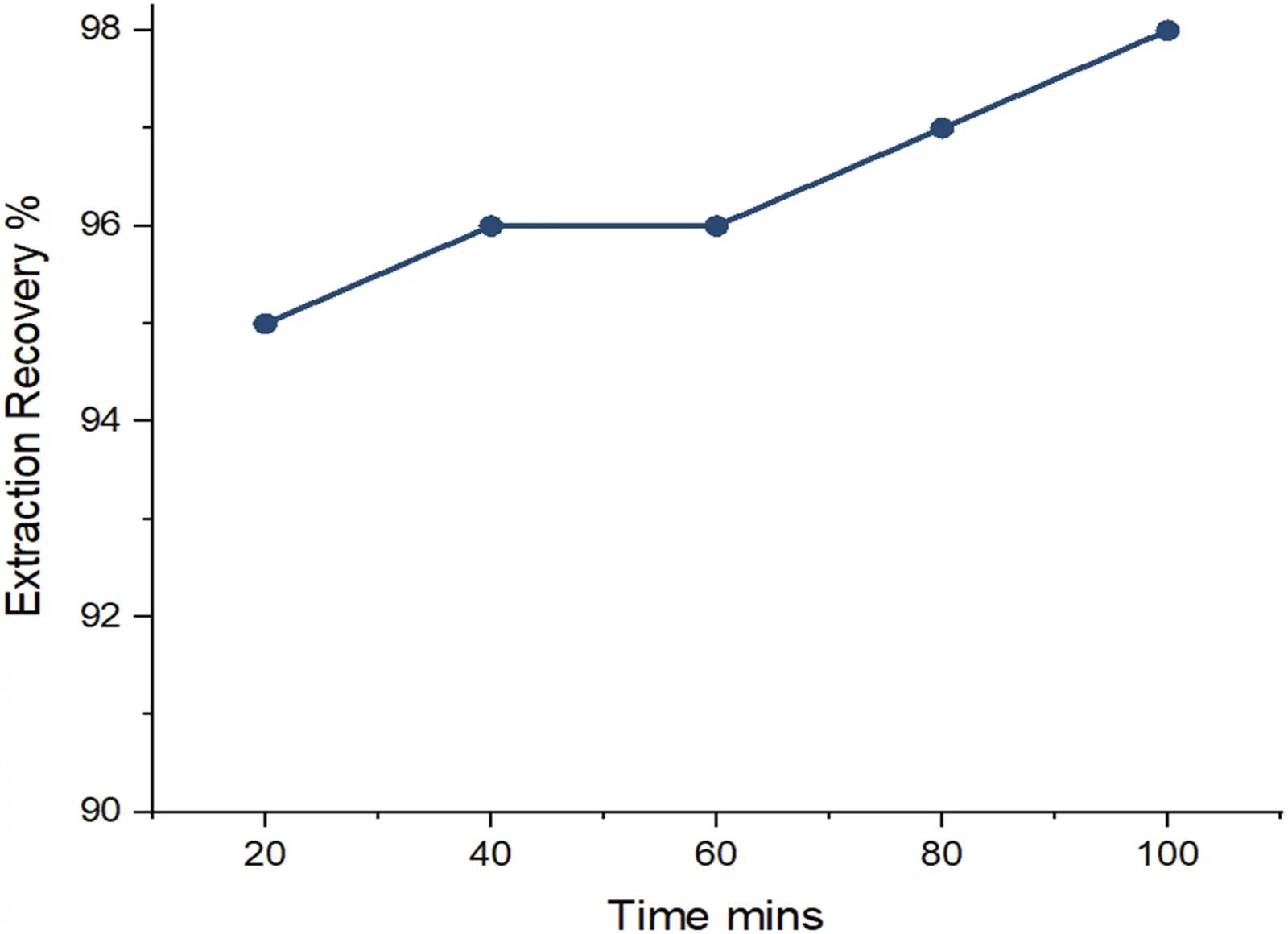
Influence of time (20–100 min) on the atrazine extraction recovery using the ZnO@CS sorbent under optimized conditions.

## 5. Kinetics studies

The pseudo-first-order (Fig. 7a) and pseudo-second-order kinetic (Fig. 7b) models were applied to understand the adsorption mechanism and determine the optimum parameters for maximum extraction of atrazine using the ZnO@CS nanocomposite cartridge. The pseudo-first-order kinetic model demonstrated the best fit to the experimental data across pH 5–9, yielding a higher correlation coefficient (*R*^2^ = 0.998) compared to the pseudo-second-order kinetic model. Further, the experimental and calculated adsorption capacity (*q*_e_) for atrazine was 14.24 mg g^−1^, indicating an excellent fit between the model and the experimental data. These results confirm that atrazine adsorption onto the ZnO@CS nanocomposite follows the pseudo-first-order kinetic model, as shown in Fig. 7a, which implies that the adsorption process is primarily controlled by the availability of active sites and pesticide concentration. The model effectively describes the rate at which equilibrium is reached with time, validating the suitability of the ZnO@CS nanocomposite for rapid pesticide adsorption.

**Fig. 7:**
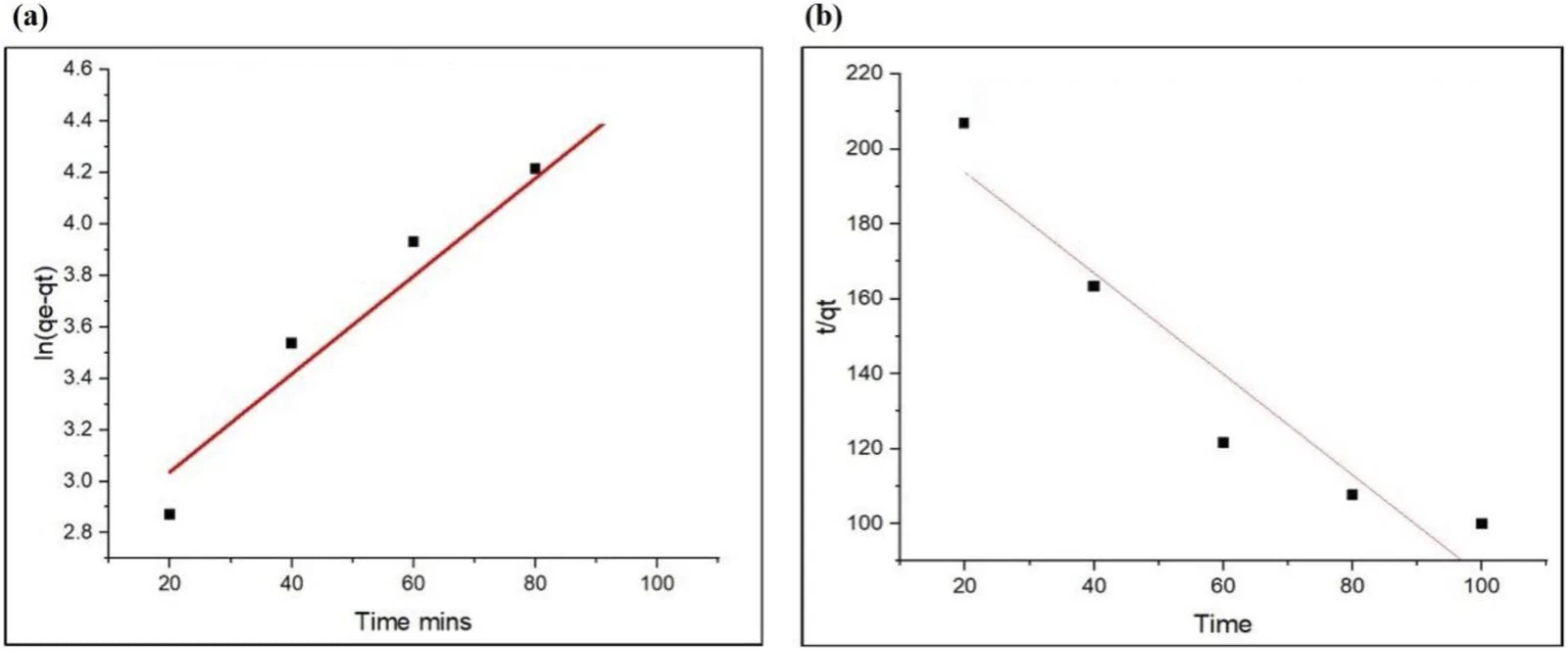
Kinetic modeling for atrazine adsorption onto the ZnO@CS nanocomposite: (a) pseudo-first-order and (b) pseudo-second-order models.

## 6. Adsorption isotherms

Adsorption isotherms were analyzed to determine the thermodynamic interactions and surface coverage mechanism of atrazine on the ZnO@CS nanocomposite. Fig. 8a and b illustrates the equilibrium isotherms fitted to the Langmuir and Freundlich models, highlighting their respective correlation coefficients (*R*^2^). The *R*^2^ value closest to unity indicates the best-fitting model. The adsorption process was better described by the Freundlich isotherm model (*R*^2^ = 0.970), which suggests that the adsorption occurs on a heterogeneous surface with sites of varying energies and allows for multilayer adsorption. On the other hand, the Langmuir model (*R*^2^ = 0.879), which assumes monolayer adsorption on a homogeneous surface, did not fit well for the ZnO@CS nanocomposite (Table S1). This is expected because the ZnO@CS nanocomposite provides a heterogeneous surface with multiple binding sites, allowing for more than one layer of atrazine molecules to be adsorbed. Overall, these results indicate that atrazine adsorption onto the ZnO@CS nanocomposite is not limited to a single layer but involves interactions with multiple active sites on the sorbent surface.

**Fig. 8:**
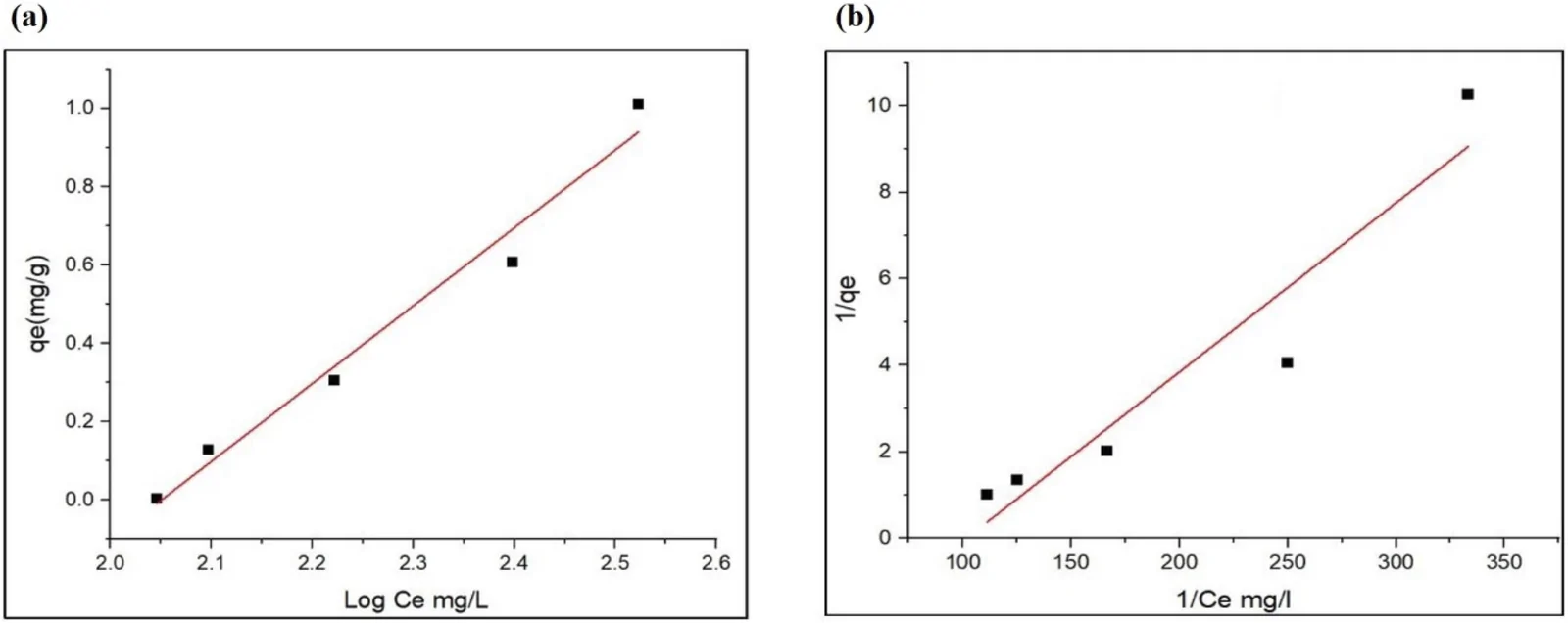
(a) Freundlich and (b) Langmuir isotherms for atrazine adsorption onto the ZnO@CS nanocomposite.

## 7. LC-MS/MS analysis of atrazine

### 7.1. Method validation

The calibration curve for atrazine showed good linearity, with a correlation coefficient (*R* = 0.996277) and a coefficient of determination (*R*^2^ = 0.992) (Fig. 9a). Single-point accuracy was assessed at three calibration levels: 50 µg L^−1^ (93.7%), 100 µg L^−1^ (108%), and 250 µg L^−1^ (97.9%), all within acceptable ranges. The LOD (3.2 µg L^−1^) and LOQ (9.7 µg L^−1^) were calculated from the calibration curve using the standard deviation of the response and the slope. The application of a weighting factor further improved precision at lower concentration levels. Overall, the method can be considered robust, sensitive, and suitable for environmental monitoring of atrazine. Fig. 9a shows the calibration curve data for atrazine standards and blanks before sample analysis using LC-MS/MS.

**Fig. 9:**
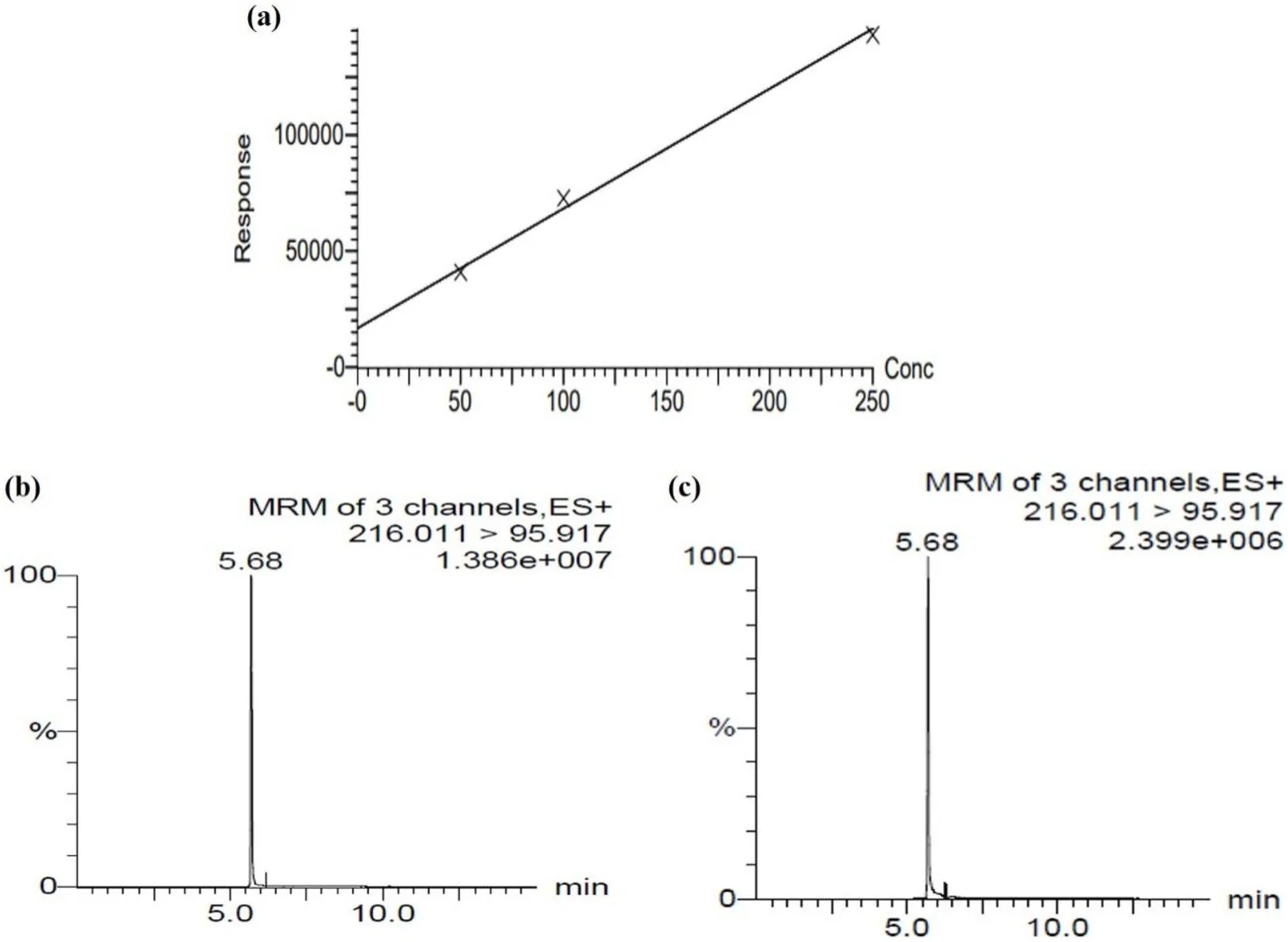
(a) Calibration curve of the atrazine standards and blanks before sample analysis in LC-MS/MS. LC-MS/MS chromatograms of synthetic water at 100 ppb (b) before extraction and (c) after extraction.

The amount of atrazine eluted from synthetic and real water samples was measured using LC-MS/MS. Following extraction, the eluted analyte (atrazine) was diluted with a 50 : 50 (v/v) mixture of DI water and methanol before being injected into the LC-MS/MS apparatus for detection and quantification in the Multiple Reaction Monitoring (MRM) channel. In the LC-MS/MS study, the starting concentration of 100 ppb resulted in an MRM transition with a precursor ion at *m*/*z* 216.01 and a product ion at *m*/*z* 95.917 in positive electrospray ionization (ES+) mode. Fig. 9b and c shows that the recorded signal intensity was 1.386 × 10^7^, demonstrating high analyte detection and confirming a considerable amount of the target compound in the sample. This finding implies that the ionization and fragmentation parameters, among other analytical conditions, were well-optimized for atrazine detection under MRM conditions, offering good sensitivity and specificity. The same MRM transition (216.01 → 95.917) was observed following extraction of the 100 µg L^−1^ solution using the ZnO@CS-loaded cartridge. The response of the chemical in the extracted sample was represented by a recorded signal intensity of 2.399 × 10^6^. This signal corresponds to a value of about 100 ppb based on the calibration curve, suggesting a recovery rate of about 90%. This demonstrates that a significant quantity of atrazine was effectively recovered at the 100-ppb level (Fig. 9c). In accordance with standard guidelines for quantifying pesticide residues, complete analytical method validation necessitates intra-day precision (repeated injections, *e.g.*, *n* = 5–6, within a single day) and inter-day precision (repeated analysis across 3 consecutive days) at multiple spiked concentration levels spanning the calibration range (*e.g.*, low, mid, and high QC levels). Accuracy is expressed as mean recovery (%) ± SD at each level. Full precision and multi-level accuracy validation of the LC-MS/MS method is identified as necessary supplementary work to support quantitative reporting of atrazine at trace environmental levels; this was outside the purview of the current study, which concentrated on establishing sorbent extraction performance.

## 8. LC-MS/MS analysis of atrazine in real water samples

The real water data show the detection and quantification of atrazine before extraction of samples analyzed using LC-MS/MS in the positive electrospray ionization mode (ES^+^), as shown in Fig. 10a and b. The MRM transition of 216.011 > 78.877 corresponds to the monitoring of a specific precursor ion (*m*/*z* 216.011), characteristic of atrazine, and its fragment ion (*m*/*z* 78.877), ensuring high specificity for identification. The recorded signal intensity of 3.827 × 10^6^ indicates a strong detection signal, suggesting a significant concentration of atrazine (100 µg L^−1^) in the agricultural wastewater sample. After extraction using the fabricated ZnO@CS nanocomposite cartridge, the agricultural field water showed 70% recovery, corresponding to 70 ppb of atrazine recovered from sugarcane field wastewater. Similarly, groundwater samples were extracted using the ZnO@CS-loaded cartridge, and atrazine was analyzed by LC-MS/MS. The real groundwater samples showed 68% extraction recovery (Fig. 10c and d). This sample was fortified with an atrazine standard to an initial concentration of 475 µg L^−1^ prior to extraction, representing a scenario of elevated point-source contamination, with a post-extraction concentration of 327 µg L^−1^. The slightly lower recovery relative to field water may be attributed to interference factors such as high electrical conductivity and total dissolved solids. In our study, a notable matrix effect was observed during atrazine recovery, decreasing to 68% for real water as compared to field water (70%) and synthetic water (98%). This reduction in extraction is consistent with matrix interference from naturally co-occurring constituents such as dissolved organic matter (*e.g.*, humic and fulvic acids), competing metal ions (*e.g.*, Ca^2+^, Mg^2+^, and Fe^3+^), and total dissolved solids, which can block active binding sites on the ZnO@CS surface or suppress hydrogen-bonding interactions. However, the present study did not include a controlled, spiked-interferent selectivity experiment in which humic acid, competing metal ions, and co-occurring herbicides (*e.g.*, simazine, propazine, or other triazines commonly applied alongside atrazine) were individually or jointly introduced at known concentrations to quantify their specific effect on atrazine recovery. Such an experiment comparing recovery in interferent-free *versus* interferent-spiked synthetic water at environmentally relevant concentrations is necessary to rigorously establish the selectivity of the ZnO@CS nanocomposite toward atrazine and to rule out non-specific adsorption. This is identified, alongside reusability testing, as a priority direction for future work to fully validate ZnO@CS as a selective and field-deployable SPE sorbent.

**Fig. 10:**
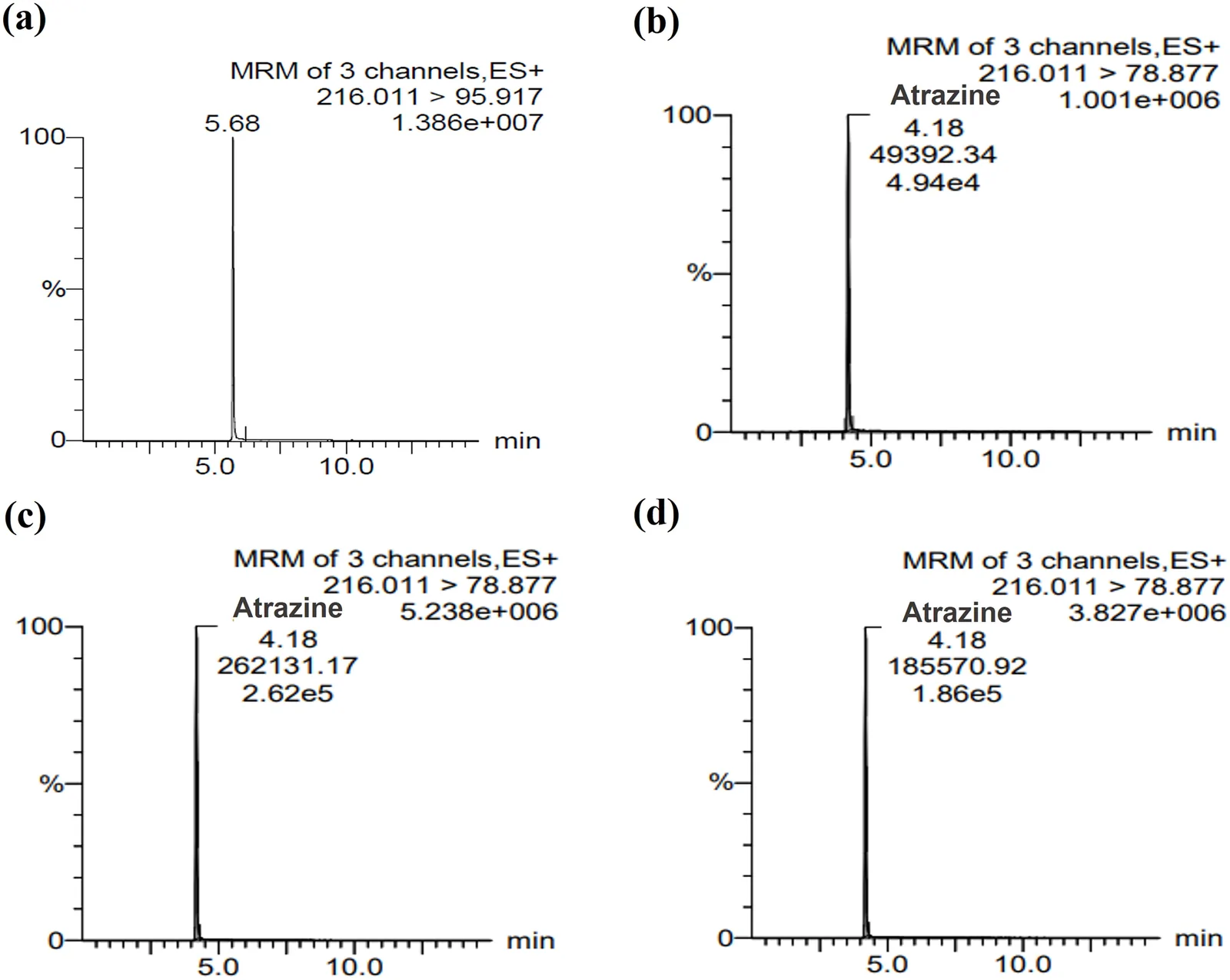
LC-MS/MS chromatograms of real field water samples (a) before and (b) after extraction. LC-MS/MS chromatograms of real groundwater samples (c) before and (d) after extraction.

## 9. Comparative extraction efficiency of the ZnO@CS nanocomposite and commercial C-18 cartridges and reported sorbents

The extraction recovery of a commercial C18 cartridge was also evaluated as a reference by passing the real agricultural field-water samples (100 µg L^−1^) through it and analyzing the extracted eluents using LC-MS/MS. The extraction recovery obtained with the C18 cartridge was 60%, as shown in Fig. 11a and b. In this study, commercially available C18 cartridges were used. Therefore, the C18 comparison is reported as an observed recovery comparison rather than as a rigorously matched performance study. In particular, equivalence of sample volume, analyte fortification, pH, flow rate, sorbent mass, conditioning, washing, elution volume, and replicate number could not be independently verified from the available records. The ZnO@CS result was 70% recovery in field water compared with 60% for C18, but the absence of replicate mean ± SD values and a statistical comparison prevents a definitive claim of statistically superior performance. Future work should repeat the comparison using fully documented and equivalent optimized conditions, multiple independent replicates, mean ± SD reporting, and appropriate statistical tests.

**Fig. 11:**
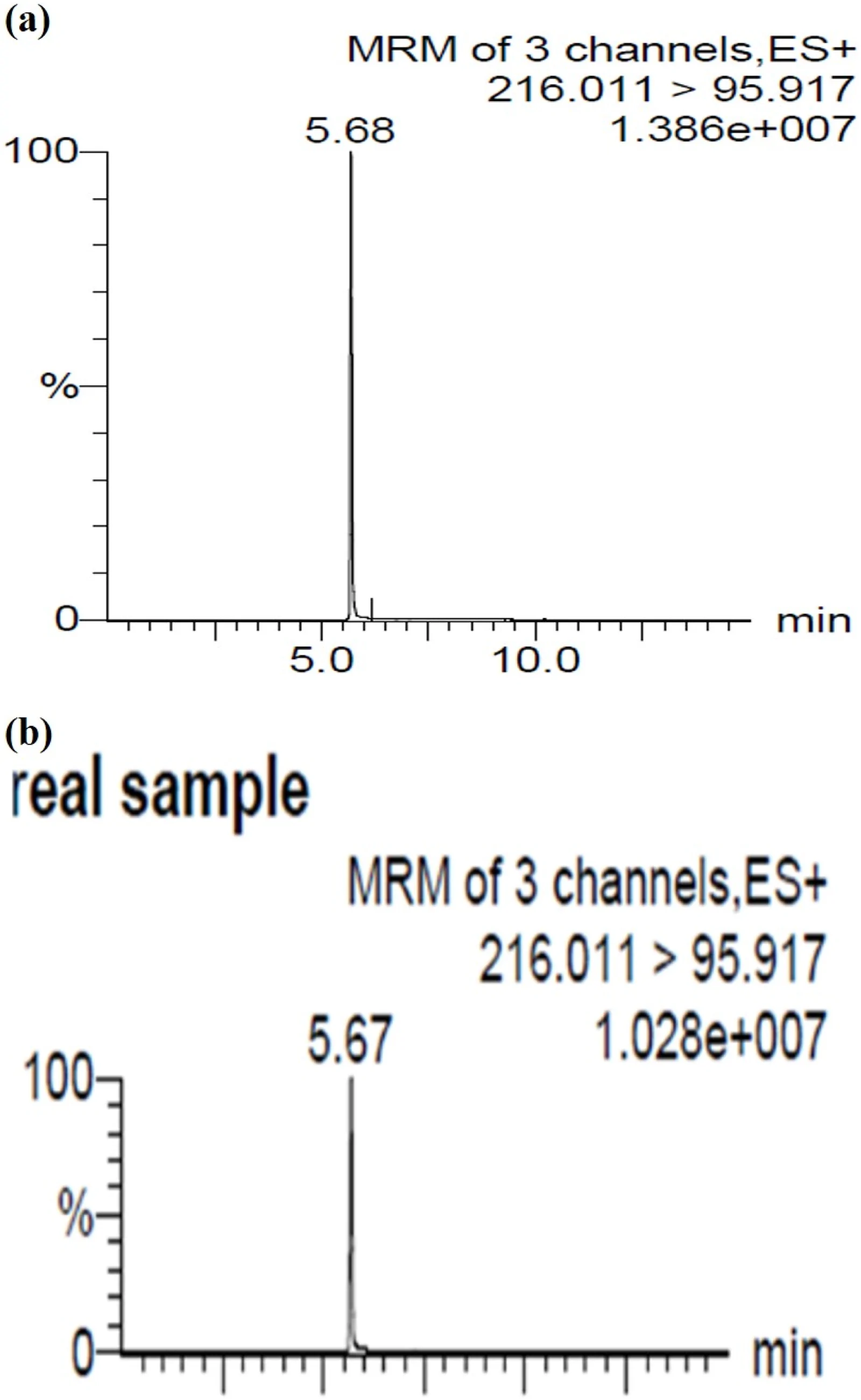
Atrazine recovery results from real water using a commercial C-18 cartridge (a) before and (b) after extraction.

Furthermore, the obtained results were compared with reported results in the literature. Table 1 summarizes the extraction performance of ZnO@CS relative to reported chitosan–metal oxide SPE and commercial C-18 cartridges. Unlike prior CS–ZnO and CS–CuO sorbents, which were primarily validated in synthetic spiked water, the ZnO@CS sorbent was additionally evaluated using real agricultural field water and groundwater, with quantification by LC-MS/MS. For the field-water samples, recovery values of 70% and 60% were obtained using the ZnO@CS and commercial C18 cartridges, respectively. However, in the absence of sufficient replicate measurements and formal statistical analysis, the difference between 70% and 60% recovery should not be interpreted as evidence of statistically superior performance of ZnO@CS.

**Table 1:** Comparative studies of the ZnO@CS nanocomposite with reported chitosan–metal oxide SPE sorbents

Sorbent	Target analyte(s)	Dosage	Optimum conditions	Recovery/adsorption capacity	Real matrix validated	Reusability tested	References
Ch–ZnO NPs	Permethrin	0.5 g	Ambient temp. and neutral pH	99% recovery	No (synthetic only)	—	43
Ch–CuO NPs	Parathion and methyl parathion	Not specified	pH 7, 25 °C, and 25 min	High removal efficiency (values not specified)	No (synthetic only)	Not reported	12
Ch–ZnO NPs	λ-Cyhalothrin, diazinon, thiophanate-methyl, methomyl, fenamiphos, and abamectin	250 mg	pH 7.0 and 25 °C	41.0–98% (analyte-dependent)	No (synthetic only)	Not reported	
C18 (commercial)	Atrazine	Standard cartridge	Standard SPE protocol	60% recovery	Yes (real field water)	Not reported	This study
TMO@CS nanocomposite	Atrazine	300 mg	pH 9, 25 °C, 100 min, and 100 mL	Synthetic: 98.0% (*q*_e_ = 14.24 mg g^−1^)	Yes (field + groundwater)	Not evaluated	This study
				Field water: 70.0% recovery			

## 10. Adsorption mechanism

FTIR studies and pH extraction experiments reveal that the adsorption of atrazine onto the surface of ZnO@CS sorbent is governed primarily by hydrogen bonding, with secondary contributions from hydrophobic interactions (Fig. 12). In addition, other parameters including ionic strength, pH, initial concentration, temperature, and surface modification also affect the extraction efficiency of atrazine. The sharp increase in extraction recovery observed up to pH 9 is attributed primarily to hydrogen bonding rather than electrostatic attraction. At pH 9, both atrazine (p*K*_a_ ≈ 1.7) and the amine groups of chitosan (p*K*_a_ ≈ 6.3–6.5) exist predominantly in their unprotonated, neutral forms. Although the nanocomposite surface carries a net negative charge under these conditions (zeta potential = −12.6 mV, Section 3.2), deprotonation of chitosan's amine groups enhances their availability as hydrogen-bond acceptors toward atrazine. This mechanism is further supported by ionic strength trials: increasing ionic concentration weakly impacts hydrogen bonding while screening electrostatic forces, confirming that electrostatic attraction plays a minor role at this pH. Additional hydrophobic interactions between atrazine's chlorotriazine ring/alkyl side chains and the organic domains of the chitosan matrix likely reinforce binding. Above pH 9, the observed decline in recovery is attributed to competitive adsorption, as excess hydroxide ions (OH^−^) compete directly with atrazine for active hydrogen-bonding sites on the sorbent surface.

**Fig. 12:**
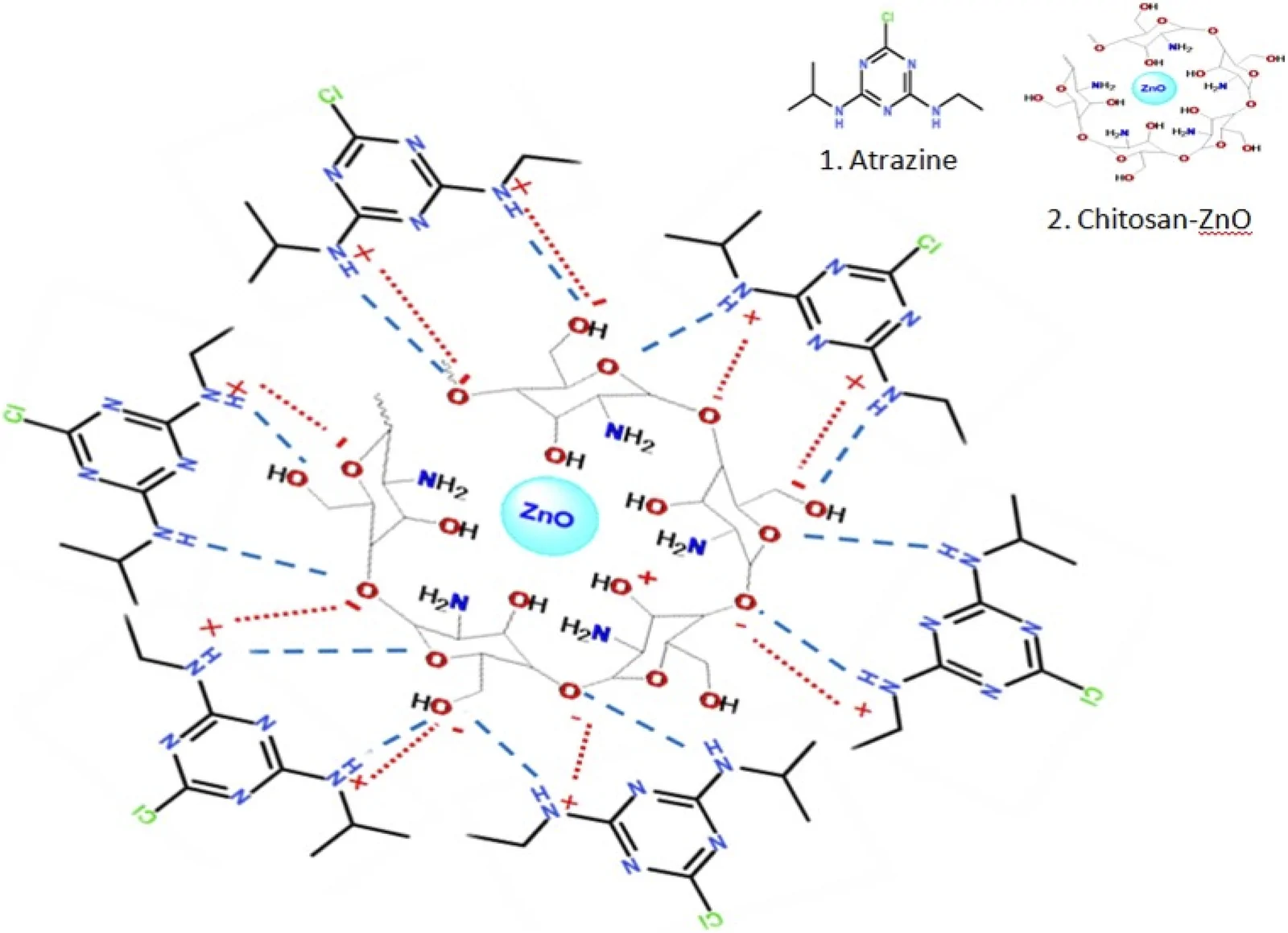
Proposed adsorption mechanism of atrazine onto the ZnO@CS sorbent through electrostatic interactions and hydrogen bonding.

## 11. Conclusion

In this study, a novel type of SPE sorbent based on the biopolymer ZnO@CS nanocomposite was synthesized using the sol–gel method and characterized by various techniques, including XRD, UV-Vis and FTIR spectroscopies, SEM/EDS, and zeta potential analysis. The as-synthesized nanocomposite sorbent was successfully applied as an SPE cartridge for extraction and recovery of atrazine pesticide from synthetic and real water samples. The prepared ZnO@CS-loaded SPE cartridge achieved a recovery of atrazine of 98% under the optimized conditions for synthetic water. More interestingly, when it was applied to real water samples, it demonstrated recoveries of ∼68% and ∼70% in groundwater and agricultural field water, respectively. Moreover, the results were compared with those obtained with a commercial C18 cartridge, which showed only 60% recovery for field water under similar conditions. Overall, this study demonstrates the potential of ZnO@CS sorbent as a low-cost chitosan–ZnO SPE sorbent for atrazine extraction from environmental water.

## Conflicts of interest

The authors declare that there are no conflicts of interest for this work.

## Supplementary Material

RA-016-D6RA06356J-s001

## Data Availability

The data supporting the findings of this work are available in this article and its supplementary information (SI). Additional datasets used and/or analyzed in this study are available from the corresponding authors upon request. Supplementary information is available. See DOI: https://doi.org/10.1039/d6ra06356j.
