# Supplementary material for: Efficient extraction of atrazine in real water samples using a biopolymer ZnO@chitosan nanocomposite-based solid-phase extraction cartridge and its quantification by liquid chromatography-tandem mass spectrometry
Source: RSC Adv. 2026 Sep 16;16(44):55334–52. doi: 10.1039/d6ra06356j (PMC13579235; doi:10.1039/d6ra06356j)
Supplement: Supplementary file 1 [file RA-016-D6RA06356J-s001.pdf]

Supplementary Information (SI) for RSC Advances.
This journal is © The Royal Society of Chemistry 2026

Supporting Data

Efficient extraction of atrazine in real water samples using a Biopolymer ZnO@Chitosan Nanocomposite-Based Solid-Phase Extraction Cartridge, and its quantification by liquid chromatography-tandem mass spectrometry

Safina Kamboh [1], Syeda Sara Hassan[1*], Saira Sidhu[1], Muhammad Raheel Bawani[2], Akbar Ali[3], Khalid Hussain Thebo[4*], Sabry M. Attia[5], Ahmed Nadeem[5]

[1]U.S - Pakistan Centre for Advanced Studies in Water (USPCAS-W), Mehran University of Engineering & Technology, Jamshoro, 76062, Pakistan

[2]Department of Mining Engineering, Mehran University of Engineering & Technology, Jamshoro, 76062, Pakistan

[3]MIIT Key Laboratory of Critical Materials Technology for New Energy Conversion and Storage, State Key Laboratory of Urban Water Resource and Environment, School of Chemistry and Chemical Engineering, Harbin Institute of Technology, Harbin 150001, PR China

[4]Department of Chemistry, Mirpur University of Science and Technology, Mirpur Azad Jammu & Kashmir, Pakistan

[5]Department of Pharmacology and Toxicology, College of Pharmacy, King Saud University, Riyadh 11451, Saudi Arabia

*Corresponding Authors: sshassan.uspcasw@faculty.muet.edu.pk (S.S.H.)**

*khalidthebo@yahoo.com (K.H.T.)**

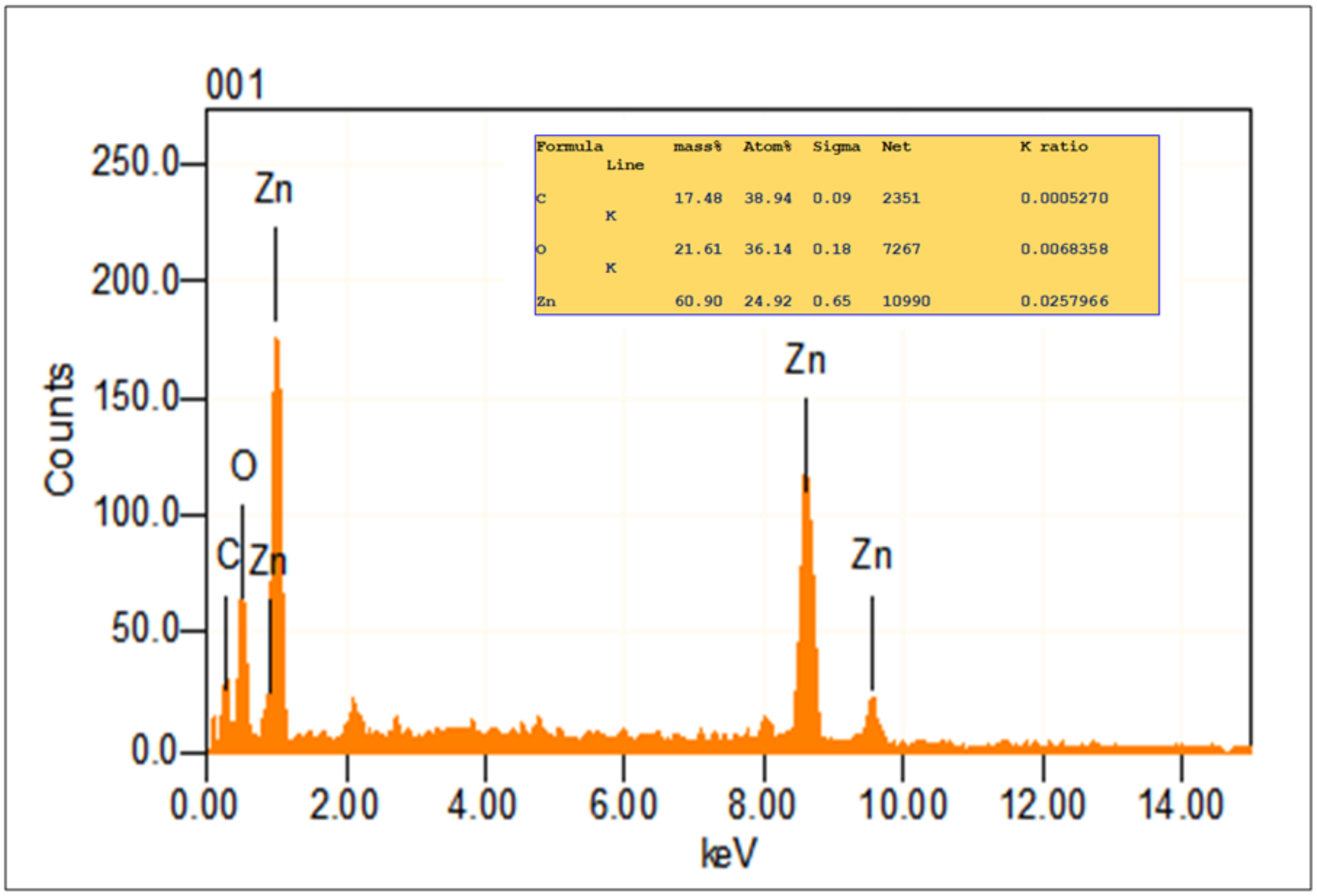

Fig. S1. EDS pattern of ZnO-decorated chitosan (ZnO@CS) nanocomposite with elements contents ratio %.

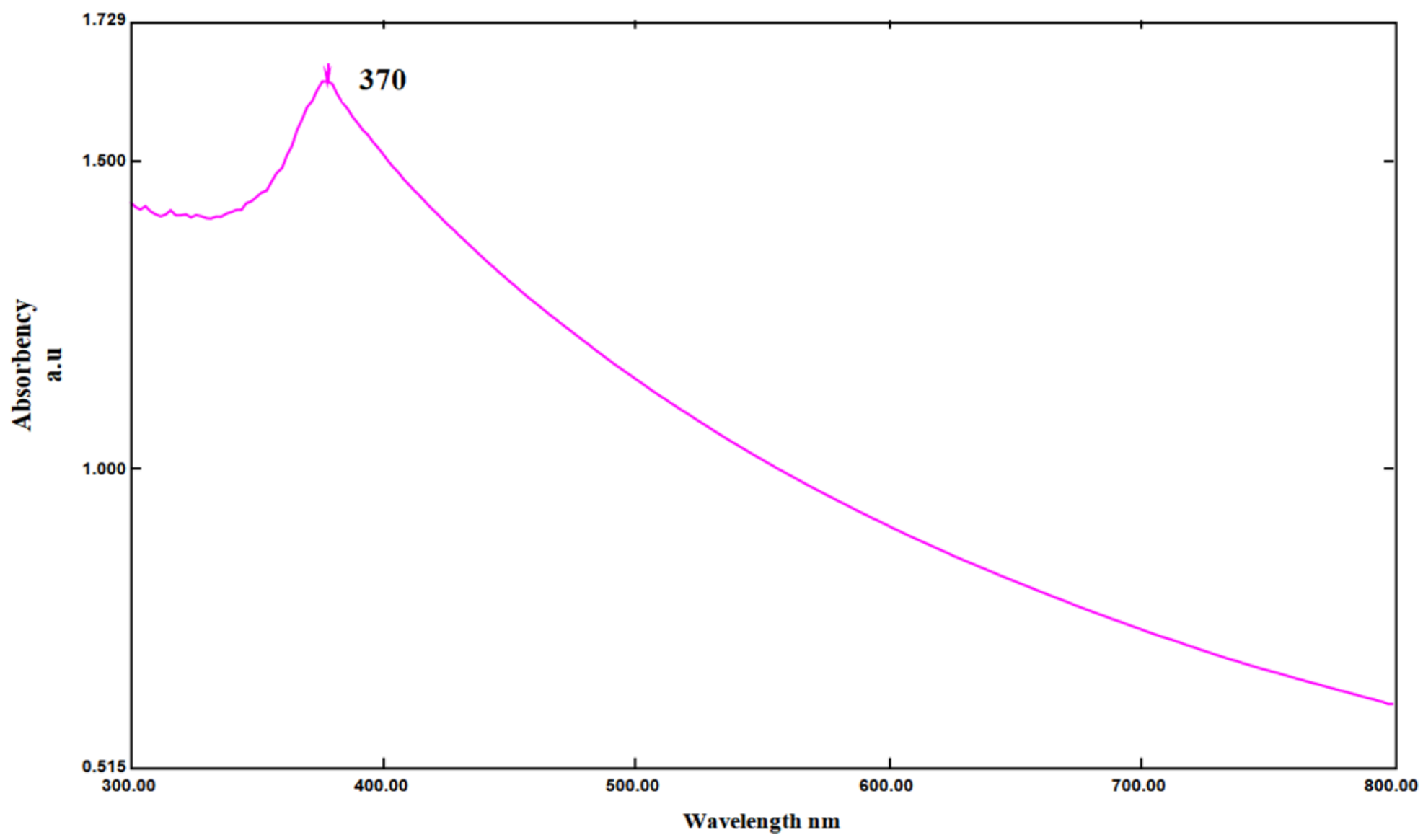

Fig. S2. UV-Visible spectrum of fabricated ZnO@CS nanocomposite at 370 nm wavelength

Table 1. Pseudo-first order, pseudo-second order kinetics parameters, Freundlich, and Langmuir Isotherm constants of atrazine adsorption onto ZnO@CS nanocomposite.

**Kinetics & Isotherms models**	**Parameters**	**Values**
Pseudo-first order kinetics model	R^2	0.998
	K1	0.00019
	**qe(mg/g)**	14.24292084
	Slope	0.01902
	Intercept	2.65626
Pseudo-second order kinetics model	R^2	0.877
	K2	25.71290967
	**qe(mg/g)**	13.9586823
	Slope	-1.34657
	Intercept	220.6998
Fruendlitch Model	R^2	0.96997
	Kf	8.35853E-05
	**1/n**	1.98893
	Slope	1.98893
	Intercept	-4.07787
Langmuir Model	R^2	0.87852
	RL	0.357468
	**KL**	-6.42532
	qmax(mg/g)	-0.25135859
	Slope	0.03912
	Intercept	-3.97838
